# Deceptive *Ceropegia sandersonii* uses an arabinogalactan for trapping its fly pollinators

**DOI:** 10.1111/nph.70144

**Published:** 2025-04-20

**Authors:** Philipp Feichtlbauer, Mario Schubert, Caroline Mortier, Christof Regl, Peter Lackner, Peter Briza, Klaus Herburger, Ulrich Meve, John W. C. Dunlop, Michaela Eder, Stefan Dötterl, Raimund Tenhaken

**Affiliations:** ^1^ Department of Environment and Biodiversity University of Salzburg Hellbrunnerstraße 34 5020 Salzburg Austria; ^2^ Department of Biosciences and Medical Biology University of Salzburg Hellbrunnerstraße 34 5020 Salzburg Austria; ^3^ Institute of Chemistry and Biochemistry Free University of Berlin Takustr. 3 14195 Berlin Germany; ^4^ Institute of Biological Sciences University of Rostock Albert‐Einstein‐Str. 3 18059 Rostock Germany; ^5^ Department of Plant Systematics University of Bayreuth Universitätsstr. 30 95440 Bayreuth Germany; ^6^ Morphophysics Group, Department of the Chemistry and Physics of Materials University of Salzburg 5020 Salzburg Austria; ^7^ Department of Biomaterials Max‐Planck‐Institute of Colloids and Interfaces Am Mühlenberg 1 14476 Potsdam Germany

**Keywords:** anti‐adhesive surface, *Ceropegia sandersonii*, fly‐trapping flower, monosaccharide composition, NMR spectroscopy, polysaccharide

## Abstract

- Many plant species have evolved surfaces that reduce insect attachment. Among such plants are deceptive trap flowers of *Ceropegia*. Their gliding zones consist of convex epidermal cells, each with a bristle‐like central protuberance and a single small liquid droplet on its tip. So far, the molecular and physical mechanisms controlling the function of these droplets are unknown.
- We analyzed the droplets of *Ceropegia sandersonii* flowers by microscopic approaches, studied how they behave when getting in contact with the feet of fly pollinators, and analyzed their chemical composition.
- The droplets contaminate the insect feet, on which they solidify. As its main component, a negatively charged polysaccharide containing a β1,3‐galactan backbone and Rha‐α1,4‐GlcA‐β1,6‐[Araf‐α1,3‐]Gal‐β1,6 side chains or truncated versions of it was identified. The chemical structure represents a rudimentary version of an arabinogalactan, which is supported by its binding to β‐d‐glucosyl Yariv reagent. Candidates of arabinogalactan proteins were identified to which the polysaccharide might be connected.
- The high amount of GlcA in the polysaccharide helps to explain the unusual physical characteristics of the droplets, like viscoelasticity and hygroscopy. We add a new function to arabinogalactans and discuss why the identified polymer is well suited for catching and temporarily trapping pollinators.

## Introduction

Around 130 million years ago, angiosperms started to emerge (Crane *et al*., 1995; Magallón *et al*., 2015), and since then, surfaces that reduce or modulate insect attachment have evolved. These surfaces often protect the plants from herbivores, while deceptive trap flowers and carnivorous plants use such surfaces to trap insects (Poppinga *et al*., 2010; Bröderbauer *et al*., 2012) for pollination purposes and to use them as a food source, respectively.

**Fig. 1:**
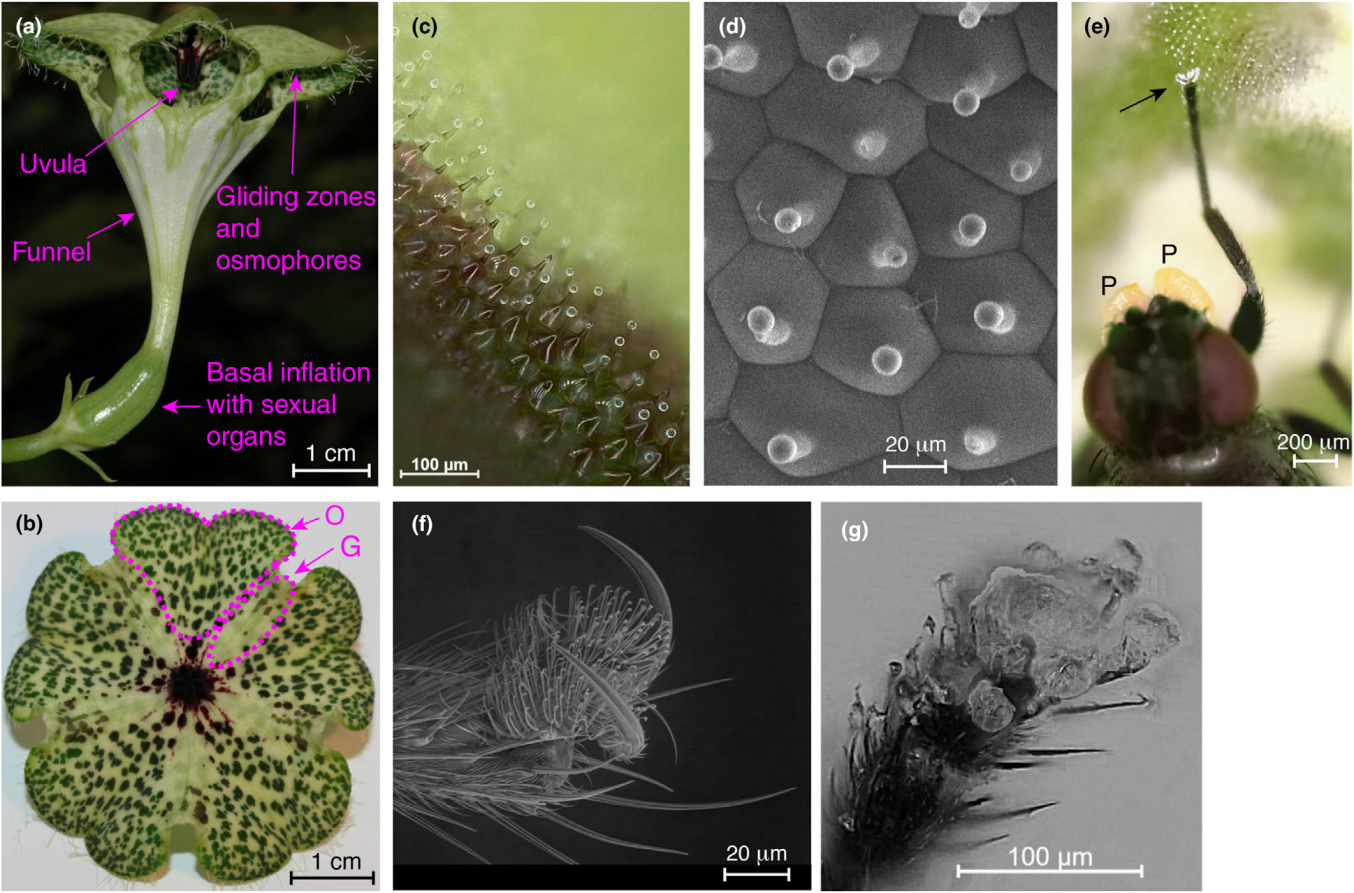
A flower of *Ceropegia sandersonii* and a fly pollinator. (a) Functional units of the flower. (b) The underside of the corolla cap consists of osmophores (O) and gliding zones (G) (see Heiduk *et al*., 2020). (c) The gliding zone with conical‐shaped epidermal cells, each with a bristle‐like central protuberance and a liquid droplet on its tip. (d) Cryo‐SEM image showing the hexagonal cells of the gliding zone. (e) A *Desmometopa* fly pollinator with solidified droplets on the tip of its front leg, and a pollinarium attached to the head (P, pollinium). (f) Tarsus of *Desmometopa* with adhesive pad and claws (scanning electron microscopy). (g) A tarsus of *Desmometopa* contaminated with solidified droplets.

Plants reduce the ability of insects to adhere to their surfaces through a variety of mechanisms, such as surface sculpturing, contamination and/or aquaplaning. Anti‐adhesion via surface texture is achieved by convex, dome‐like, papillae‐like or tabular‐shaped cells that result in roughness (Poppinga *et al*., 2010). Such an arrangement of cells on the plant surface prevents claw interlock, by not providing the adequate edges or ridges that insect claws can successfully lock on to (Juniper *et al*., 1989; Vogel & Martens, 2000). Furthermore, these surfaces also act to greatly reduce the overall contact area for insect footpad adhesion thus reducing possible adhesion forces (Gorb & Gorb, 2002; Gorb *et al*., 2005; Poppinga *et al*., 2010). An anisotropic arrangement of lunate cells or small trichomes can also achieve the same effect (Juniper *et al*., 1989; Gaume *et al*., 2002; Poppinga *et al*., 2010; Gorb & Gorb, 2011; Bauer *et al*., 2012). While all these surface types reduce the contact area on a microscopic scale, the formation of three‐dimensional epicuticular waxes reduces the contact area on a nanoscopic scale (Gorb, 2001; Gorb & Gorb, 2017). This is achieved by radial ridges (see Bohn & Federle, 2004) on plant surfaces by superimposed wax crystals or cuticular folds (Bohn & Federle, 2004; Prüm *et al*., 2012; Surapaneni *et al*., 2021) among others. Anti‐adhesive properties via contamination are achieved by interfering with the adhesive properties of the insect footpads (Knoll, 1914; Poppinga *et al*., 2010). Here epicuticular wax layers display either filamentous or tubular crystals (Federle *et al*., 1997; Gaume *et al*., 2004; Borodich *et al*., 2010) or platelets (Juniper *et al*., 1989; Gaume *et al*., 2004; Gorb *et al*., 2005; Purtov *et al*., 2013), both of which may easily break off the plant surface and attach to the insect's adhesive footpads, thus contaminating the foot surface. Another strategy to reduce insect attachment is to soak up the adhesive liquids that insects produce on their footpads through a highly porous wax layer present on the plant epidermis (Gorb *et al*., 2017; Gorb & Gorb, 2017). Footpad adhesion can also be disturbed via films of water or fatty oils on the plant surface (Knoll, 1922; Bohn & Federle, 2004). These can either reduce adhesion directly or can contaminate the feet of the insect. Some plants may even combine more than one of the above described features to boost their anti‐adhesive properties. Among such plants are the deceptive kettle trap flowers *Ceropegia stapeliiformis* Haw. (Vogel, 1965) and *Ceropegia sandersonii* Decne. ex Hook. f. (Vogel, 1961; Heiduk *et al*., 2020) that have corolla lobe epiderms that are equipped with convex outer walls topped by a papilla secreting a single small liquid droplet on its tips. When an insect footpad contacts a droplet, it immediately attaches to the pad making the footpad nonfunctional (Vogel, 1965). The corolla of *Ceropegia* (Apocynaceae) trap flowers constitute a kettle at its base (ostiolum), followed by the tube and free corolla tips which are, however, often brought together at the tips again as in *C. sandersonii*, where the fused tips form a parachute‐like structure that ‘caps’ the corolla (Vogel, 1965; Fig. 1a,b). In this species, kleptoparasitic fly pollinators are attracted to the flowers by their scents mimicking prey of the flies (honey bees) (Heiduk *et al*., 2016). The scents are produced by the flowers in osmophores found in specific regions on the underside of the corolla cap (Fig. 1a,b). In‐between the osmophores are the gliding zones (Fig. 1b). Centrally, the purple‐black ‘uvula’ (Fig. 1a) protrudes into a cone‐like structure, representing the centrally fused ends of the five gliding zones of the cap (cf. Heiduk *et al*., 2020).

Pollinators land on the flower, enter the corolla through one of five openings and, when moving to a gliding zone, slip and slide down the funnel to finally get temporarily trapped in the kettle, where downward‐pointed hairs prevent them from escaping (Vogel, 1961). After pollination, the hair‐like trapping mechanism becomes nonfunctional, allowing the flies to escape and potentially pollinate another flower (Vogel, 1961).

This complex pollination mechanism has already been studied from some key perspectives, with most recent research focusing on the development of the flowers (Heiduk *et al*., 2020) and on the chemical nature of the scents produced by the osmophores and their importance in attracting and fooling the pollinators (Heiduk *et al*., 2016). Many questions remain about the chemical and physical aspects of the droplets in the gliding zone. It seems that the droplets somehow contaminate the pollinators feet (Vogel, 1965). In order to better understand the molecular and physical mechanisms that control the function of the droplets, we studied in detail how the liquid droplets found in the gliding zones of *C. sandersonii* flowers behave when getting in contact with the footpads of fly pollinators. Due to the metastable nature of these droplets, which made a detailed physical characterization extremely difficult, we focused mainly on characterizing the chemical composition of the droplets, which may assist further in understanding function. Droplets were collected to analyze their chemical composition. Our data revealed that the droplets consist of a water‐soluble arabinogalactan polymer that solidifies on the feet of pollinators, interfering with the adhesive properties of the insect footpads by contaminating them.

## Materials and Methods

### Plant and animal material

Four plant individuals of *Ceropegia sandersonii* Decne. ex Hook f., obtained from a commercial breeder, were used for the experiments. The plants were cultivated in the glasshouses of the Botanical Garden of the Paris Lodron University of Salzburg. Pollinating *Desmometopa* (Milichiidae) flies were collected from the flower kettles of the four *C. sandersonii* plants.

### Microscopy

Stereo light microscopy (Leica S8APO, equipped with a MC170HD camera; Leica S6D; Leica, Wetzlar, Germany) was used to image the gliding zone as well as to provide imaging during the extraction of the liquid droplets and the interaction of the droplets with the flies.

Based on the light micrographs, small pieces of the gliding zone were isolated with a razor blade and mounted on a cooled sample stage of an environmental scanning electron microscope (ESEM) (FEI‐Quanta 600 FEG, FEI electron optics, Eindhoven, the Netherlands). The cooling stage consisted of a Peltier element, allowing fast control of the temperature. The heat of the other side of the Peltier element was cooled by a water system, operated at 2–4°C. A gliding zone sample was kept at a temperature of *c*. 2–3°C; the ESEM was pumped to a pressure of *c*. 7 Torr, and water vapor was used as an imaging gas to visualize the gliding zone in a native, hydrated state at an acceleration voltage of 10 keV. By increasing the pressure of the water vapor, a water film covered the sample surface. Pressure reduction was used to dry the sample.

To back up the ESEM experiments and to get more data about the droplet size, gliding zone samples were frozen in liquid nitrogen and afterwards cryo‐transferred into a cryo‐SEM (JEOL 7500F; JEOL Ltd, Tokyo, Japan). The samples were freeze‐etched and afterwards imaged under cryo conditions at 2 keV acceleration voltage.

### Interaction of fly legs with the gliding zone

Flies collected from the kettle traps of *C. sandersonii* were temporarily sedated using carbon dioxide (CO_2_) gas and observed under a stereo microscope to check for foot contamination. Only flies without obvious contamination were used for the following experiments. In total, 15 temporarily sedated flies (two to four flies at a time, depending on availability) were put on the gliding zone of an upside‐down corolla cap (i.e. with the gliding zone facing upwards) and observed after waking up from sedation. The corolla cap with the flies was covered by a glass Petri dish to avoid the flies escaping. Additionally, legs of freeze‐killed flies were used to brush them over the gliding zones. Following the experiments, the legs of the flies were observed under the stereo microscope described above.

### Sampling of droplets

To obtain samples for chemical analyses, droplets were collected using two approaches. The first approach used glass capillaries (Marienfeld 100 × 1.5 mm), pulled using a NARISHIGE PC‐10 capillary puller (Amityville, NY, USA) to form capillaries with an outer tip diameter of 10–20 μm, to collect single droplets. Before collection, the tips of the capillaries were sealed by moving them quickly through a Bunsen burner, giving them a smooth and round tip. For droplet collection, the tip of such a glass capillary was brought in contact with a single droplet using a Syntech MP‐15 micromanipulator (Syntech, Kirchzarten, Germany). Upon contact, each droplet solidified immediately on the tip of the capillary. This procedure was repeated for 20–40 droplets, before dissolving the accumulated solidified droplets in ultrapure water. Overall, *c*. 40 of such solidified droplets were collected from each of 20 flowers.

In the second approach, the droplets were washed off from the gliding zone to obtain higher amounts of droplet solution. A 200‐μl pipette was used to apply a small amount of water (< 70 μl) onto the gliding zone. The water dissolved the droplets before it was pulled back into the pipette. The surface tension was sufficient to hold the water drop in place, and the hydrophobic surface of the gliding zone allowed retrieving nearly all of the water back into the pipette. The droplets of the gliding zone of 5–10 flowers were dissolved in 400 μl ultrapure water, and overall, *c*. 300 flowers of the four individual plants were sampled using this approach. To obtain negative control samples, we washed off the surface of the osmophore, which neighbors the gliding zone of the corolla cap (Fig. 1b; and Heiduk *et al*., 2020).

### Nuclear magnetic resonance spectroscopy

Nuclear magnetic resonance (NMR) spectra were measured on a Bruker Avance III HD 600 MHz spectrometer with a QXI room temperature probe (both Bruker Biospin, Ettlingen, Germany) at 298 K using D_2_O (100.0% D from Armar Euope, Leipzig, Germany) as solvent. The lyophilized samples (original sample, hydrolysate or cleaving products of polymer; refer to the subsequent section) originating from 50 flowers each were dissolved in 270 μl D_2_O (100.0% D; Armar Europe, Leipzig, Germany) and measured in 5‐mm NMR Shigemi tubes. Chemical shift assignment was achieved with 2D ^1^H–^1^H TOCSY (total correlated spectroscopy, mixing times of 80 and 12 ms), 2D ^1^H–^1^H COSY (correlated spectroscopy), 2D ^1^H–^13^C HSQC (heteronuclear single quantum correlation), 2D ^1^H–^13^C HMBC (heteronuclear multiple‐bond correlation) and ^1^H 1D spectra, using the Bruker pulse sequences mlevphpp, cosygpmfphpp, hsqcedetgpsisp2.2, hmbcgplpndprqf and zg30, respectively. Spectra were processed with Topspin 3.6.2 (Bruker) and analyzed with Sparky 3.115 (T. D. Goddard and D. G. Kneller, SPARKY 3, University of California, San Francisco, CA, USA). For referencing, DSS (2,2‐dimethyl‐2‐silapentane‐5‐sulfonic acid; Armar Europe) was added to the samples after measuring all other spectra. A 1D ^1^H experiment was performed for referencing the proton chemical shift. Carbon dimensions were referenced according to the IUPAC‐IUB recommended chemical shifts referencing ratio of 0.251449530 (Markley *et al*., 1998).

### Sugar analysis by high‐performance anion‐exchange chromatography/pulsed amperometric detection (HPAEC‐PAD)

The *Ceropegia* droplet material was hydrolyzed in 2 M trifluoroacetic acid at 121°C for 1 h. The samples were dried in a vacuum centrifuge and redissolved in 200 μl ddH_2_O. Sugars were separated on a CarboPac PA20 column (3 × 50 mm precolumn, 3 × 150 mm separation column; flow rate 0.45 ml min^−1^) using a Dionex ICS3000 chromatography system with pulsed amperometric detection (Thermo Fisher Scientific). Samples were separated with two different gradients (Supporting Information Table S1).

Quantification of monosaccharides was performed using authentic commercially available standards. An unknown peak (refer to the subsequent section), which eluted similarly to GlcA, was different from GlcA and 4‐O‐methyl GlcA as proven by co‐chromatography with authentic standards.

### Enzymatic cleavage

*Ceropegia* droplet material (*c*. 8 μg) was incubated with a glucuronidase from *Fusarium oxysporum* Schlechtend. for 3 h at room temperature using described conditions (Kondo *et al*., 2021b). The digest was terminated by heating to 95°C for 10 min. The solution was transferred to a Microcon tube filter (UItracel YM10; 10 kDa cutoff; Millipore) and centrifuged until the membrane filter was almost dry. A 100 μl of H_2_O was added twice to remove residual low molecular weight compounds from the concentrate. Both the filtrate and the retentate were collected and lyophilized.

### Arabinogalactan test with Yariv reagent

We incubated three droplet samples with β‐d‐glucose Yariv (Biosupplies, Vilnius, Lithuania), binding selectively to type II arabinogalactans (AGs), using the method described in Bio‐Protocols (Lamport, 2013). Gum Arabic (Biosupplies; 10 and 20 μg) was used for positive quantification of AGs. We used α‐d‐galactose Yariv as a negative control, as recommended by the supplier.

### Comprehensive microarray polymer profiling

The glycan microarray analysis (Moller *et al*., 2007) of the *Ceropegia* polymer was done as described in detail before (Kračun *et al*., 2017). Arrays were printed as distinct dots onto nitrocellulose membranes with an ArrayJet Sprint microarray printer (ArrayJet, Roslin, UK) and probed with 46 cell wall probes, including antibodies and carbohydrate binding modules (Table S2). Probing was performed in duplicates. For controls, primary antibodies were heat inactivated before use.

### Genome/transcriptome analyses

Total RNA was extracted from the osmophor and the gliding zone, using the Trizol method, and sent to Novogene for RNAseq (30 million reads, 2 × 150 bp). The Trinity assembled transcripts were translated with transeq to protein sequences for all possible reading frames. The longest continuous protein sequence was extracted for the proteomics analysis. Transcript annotation was performed by Blastx against UniProt. A proteomics experiment was performed on an aliquot of the gliding zone wash off to confirm the presence of proteins corresponding to detected transcripts. First, the proteins in the sample were enzymatically digested into peptides for analysis using trypsin. Next, the generated peptides were separated using reversed‐phase HPLC on a C18‐based nano‐HPLC column and subsequently analyzed by high‐resolution tandem mass spectrometry (HPLC‐MS^2^). A detailed method for the identification of the potential protein component of the *Ceropegia* arabinogalactan protein (AGP) is found in the Methods S1.

## Results and Discussion

### Morphology of gliding zone and interaction with fly legs

**Fig. 2:**
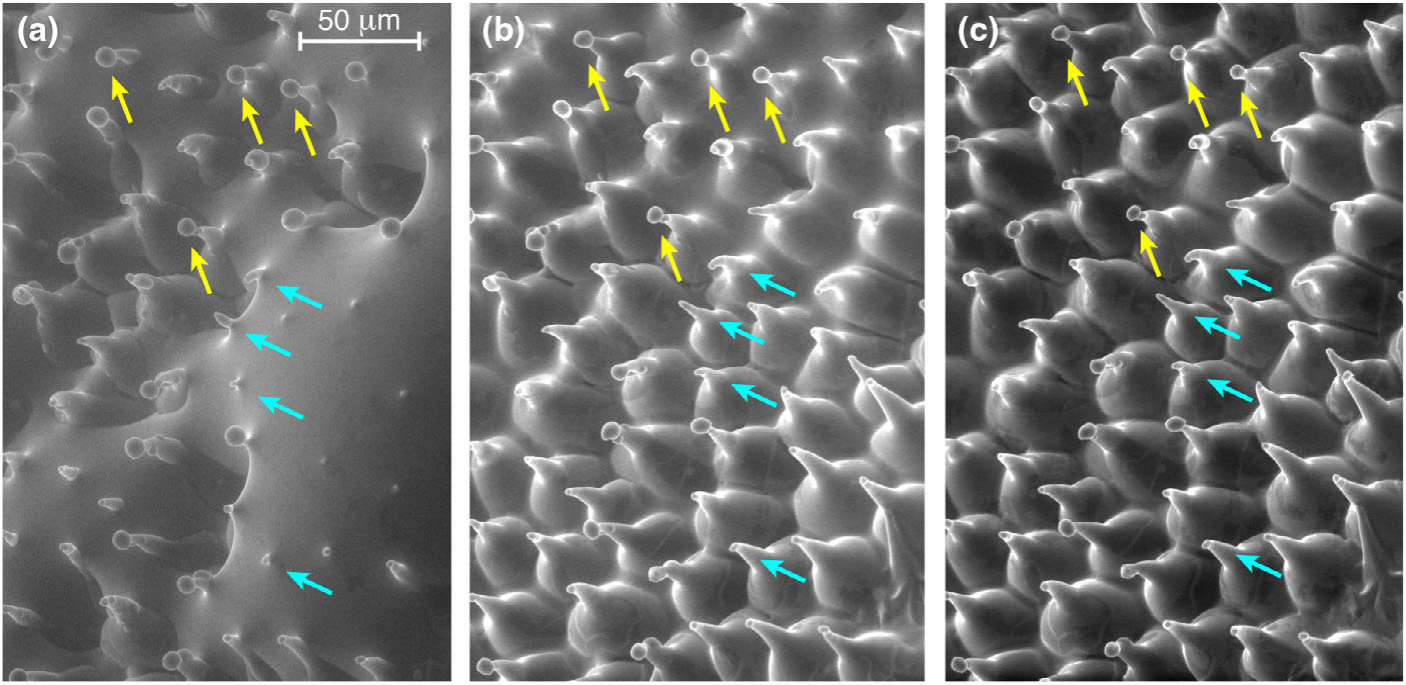
Environmental scanning electron microscopy of a section of the gliding zone of *Ceropegia sandersonii* measured at 7.25 Torr (a), 5.45 Torr (b) and 4.75 Torr (c). The gliding zone is partly covered by a film of water in (a, b). Yellow arrows indicate droplets not in contact with water. Cyan arrows indicate cells from which the droplets were removed by the water.

The gliding zone on the underside of the corolla cap (morphologically the upper side of the corolla tips, Fig. 1a,b) consists mainly of pentagonal and hexagonal cells that have a bristle‐like central protuberance (Fig. 1c,d). During anthesis, which lasts a few days, each of these elongations bears a spherical liquid droplet, with a diameter of *c*. 10 μm, on its tip (Fig. 1c,d). The droplets remained stable on the elongations, even at low chamber pressures in the ESEM, which would normally lead to drying of pure water droplets. By contrast, the droplets were easily dissolved and washed off in water (Fig. 2). When *Desmometopa* fly pollinators (*n* = 15) were placed on the gliding zone of an upside‐down flower cap, they all removed droplets from the gliding zone while trying to get foothold. The droplets quickly (less than a second) formed solid aggregations on their feet (Fig. 1g). Still, flies managed to roam around on the flower cap as long as the gliding zone was flat. Flies were not able to climb on steep ridged gliding zones and slid off, thus highlighting gliding zone function.

Solid aggregations on fly legs, especially the footpads (Fig. 1e), were also observed when manually brushing an insect leg several times over the gliding zone (Fig. 1f). Solidification happened within a fraction of a second. Similar observations were made in preliminary experiments when droplets were touched with needles or glass capillaries and even hair, with droplets wetting the surface and solidifying almost instantly independent of the surface used. These observations and experiments confirm that the droplets contaminate the feet, preferably the tarsi, of the flies (Fig. 1g). Compared to other plants with this strategy (as stated in the Introduction), however, the matter involved in contamination changes its state, that is, from liquid to solid. Compared to solid materials, such as epicuticular wax platelets (Juniper *et al*., 1989; Gaume *et al*., 2004), the liquid droplets might more easily contaminate a leg of a fly that moves upside down on the bottom side of the corolla cap of a *C. sandersonii* flower. After being trapped in the kettle of the flowers, flies might easily remove the solidified droplets from their legs and freely move in the kettle, an important prerequisite to performing pollination behaviors while being trapped. Removal of the solidified droplets from the legs will also help the flies escape from the flower and potentially export pollen to another flower of *C. sandersonii*. Along these lines, most flies collected from the kettle of the flowers did not have solidified droplets on their legs, demonstrating the temporary nature of the contaminant. Some plants, such as deceptive *Arum nigrum* Schott, a species pollinated by flies and beetles (Knoll, 1926; Gibernau *et al*., 2004), contaminate the legs of their pollinators with lipophilic, nonspherical oil droplets available on each cell of the gliding zone (Knoll, 1926). It is unknown whether pollinators can remove the oil from their legs while being trapped in the inflorescence and before leaving the inflorescence to potentially visit another one.

### Preliminary chemical analysis of the droplets

**Fig. 3:**
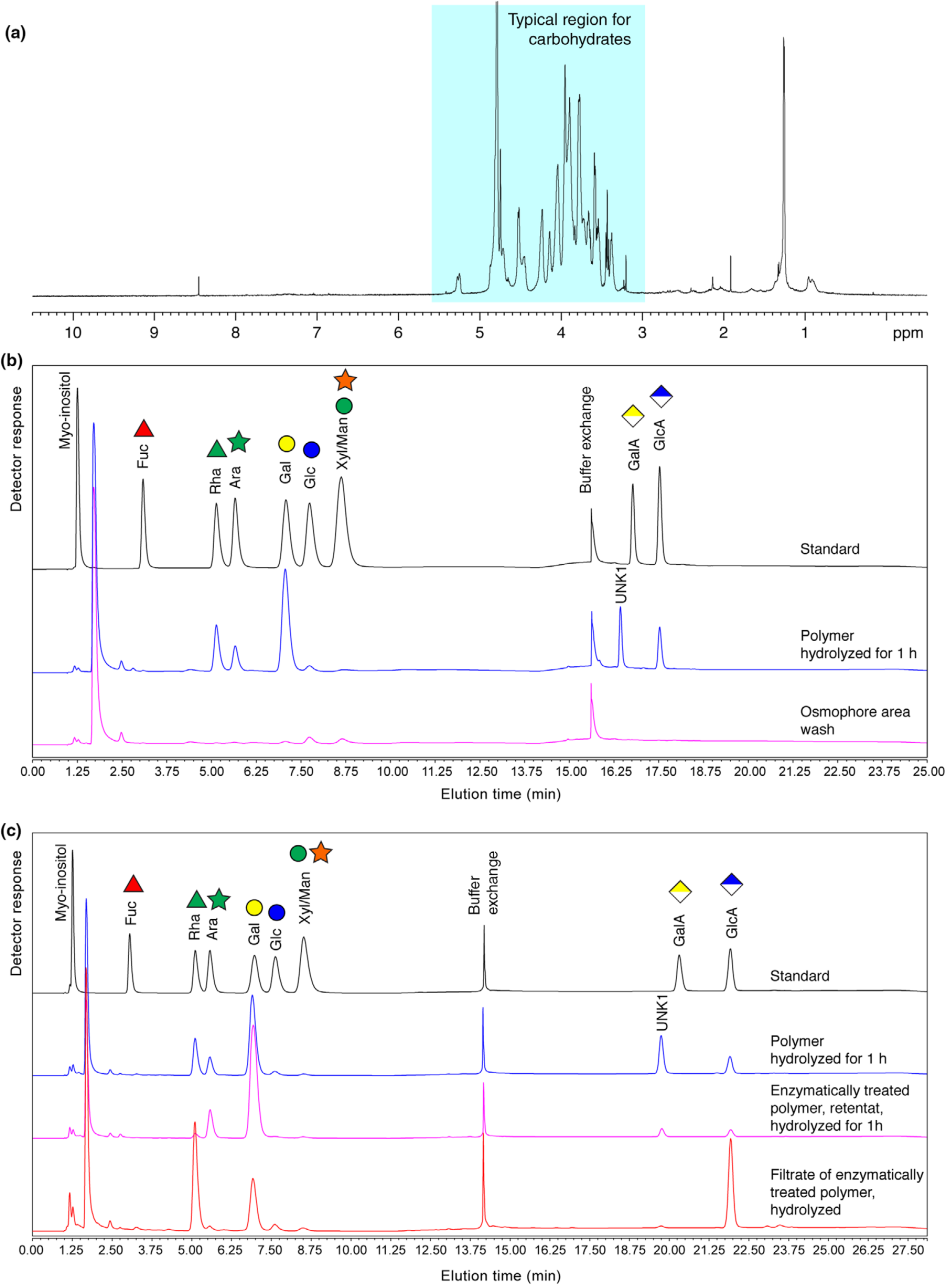
Composition of the secreted droplets of *Ceropegia sandersonii* analyzed by ^1^H nuclear magnetic resonance (NMR), and monosaccharide composition of the original droplets and the hydrolyzed polymer by high‐performance anion‐exchange chromatography coupled with pulsed amperometric detection (HPAEC‐PAD). (a) ^1^H NMR spectrum of lyophilized droplets of 50 flowers dissolved in D_2_O. (b) HPAEC‐PAD of the polymer washed off the gliding zone or the osmophore zone of a flower in comparison with monosaccharide standards of fucose (Fuc), rhamnose (Rha), arabinose (Ara), galactose (Gal), glucose (Glc), mannose (Man), xylose (Xyl), galacturonic acid (GalA) and glucuronic acid (GlcA). Polymer samples were hydrolyzed in 2 M TFA for 1 h, dried in a vacuum centrifuge to dryness. The samples were separated using gradient 1 (see the Materials and Methods section). The hydrolyzed polymer from the gliding zone is composed of Gal, Rha, Ara, GlcA and an unknown sugar eluting slightly earlier than GalA (blue curve), whereas the sample from the osmophore zone shows no sugar signal (pink curve at bottom). The chromatogram of the reference monosaccharide standard solution is shown in the top (black) line together with standard symbols for the monosaccharides. (c) In order to identify the nature of the unknown sugar peak, we incubated the polymer with a recombinant glucuronidase from *Fusarium* (Kondo *et al*., 2021a) and separated the sample by filtration on a 10 kDa membrane. The hydrolysate of the filtrate (lower curve in red) contained mainly GlcA and Rha along with some Gal as revealed by separation with gradient 2. The high molecular retentate hydrolyzed to Gal and Ara but the unknown peak disappears (pink curve). The hydrolysis of the polymer (blue line) and reference sugars (black line) are shown on top for comparison.

Nuclear magnetic resonance spectroscopy (one‐dimensional ^1^H spectrum) of a lyophilized original sample revealed that a carbohydrate is the main component of the droplets (Fig. 3a). The broad line widths are typical for a long polysaccharide.

### Composition of the polysaccharide

**Fig. 4:**
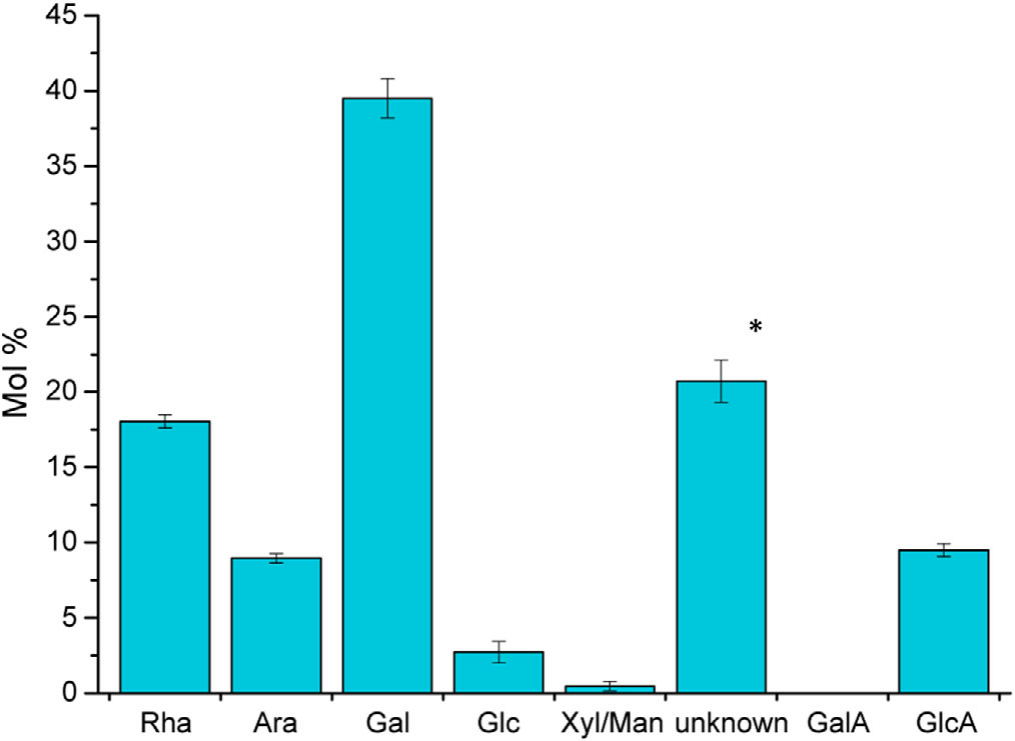
Sugar analysis (mean ± SD) of the hydrolyzed polymer of *Ceropegia sandersonii*. The samples obtained by collecting droplets with a micromanipulator (*n* = 1) or by washing off the gliding zone (*n* = 2) resulted in a highly similar composition as indicated by the small SD. Sugars were separated with gradient 1 (see the Materials and Methods section). The monosaccharides rhamnose, arabinose, galactose, glucose, xylose, mannose, galacturonic acid and glucuronic acid are abbreviated as Rha, Ara, Gal, Glc, Xyl, Man, GalA and GlcA, respectively, according to standard nomenclature. The unknown peak eluted from the HPLC column close to uronic acids. Therefore, we used as an estimation the response factor for GlcA for quantification (indicated by ‘*’).

To determine the composition of the polysaccharide, three *Ceropegia* samples were analyzed by high‐performance anion‐exchange chromatography with pulsed amperometric detection (HPAEC‐PAD), one obtained by collecting droplets with a micromanipulator, the other two obtained by washing off the gliding zone. In agreement with the initial NMR data, we did not find any signal for monosaccharides, pointing to a polymer. To analyze the monosaccharide composition, the polysaccharide was hydrolyzed in 2 M trifluoracetic acid and analyzed using HPAEC‐PAD (see the Materials and Methods section), which revealed galactose (Gal), rhamnose (Rha), arabinose (Ara), glucuronic acid (GlcA) and an unknown compound (Fig. 3b) as major constituents. A quantification of the hydrolyzed sugars showed that the ratio of sugars among all three samples is highly similar, confirming that both sampling methods lead to the same overall result (Fig. 4). Our first assumption, based on the elution conditions of the unknown compound, that it would be 4‐Me‐GlcA was proven wrong, since the signal did not match with an authentic reference of 4‐Me‐GlcA.

One of the hydrolysates was also analyzed by one‐ and two‐dimensional NMR spectroscopy, and the signals were matched to comparable spectra of monosaccharide references. The 1D spectra are shown in Fig. S1. Readily confirmed were Gal, Ara and Rha. The analysis was complicated not only by separate signals for the α‐ and β‐form of each monosaccharide, but also by the absence of GlcA and instead the presence of two forms of GlcA‐γ‐lactone and the presence of an unknown component. Overall, the NMR analysis was consistent with the HPLC data, but additionally indicated the presence of an unknown disaccharide (refer to the subsequent section) and GlcA‐γ‐lactone. As shown previously, GlcA‐γ‐lactone is built under acetic conditions during hydrolysis from GlcA (Uhliariková *et al*., 2021), suggesting that this lactone is, in contrast to GlcA, not a constituent of the polymer. The chemical shift assignment of the unknown compound fits the previously described disaccharide GlcA‐β1,6‐Gal (Fig. S2; Table S3).

### Antibodies binding to *Ceropegia* polymer

**Fig. 5:**
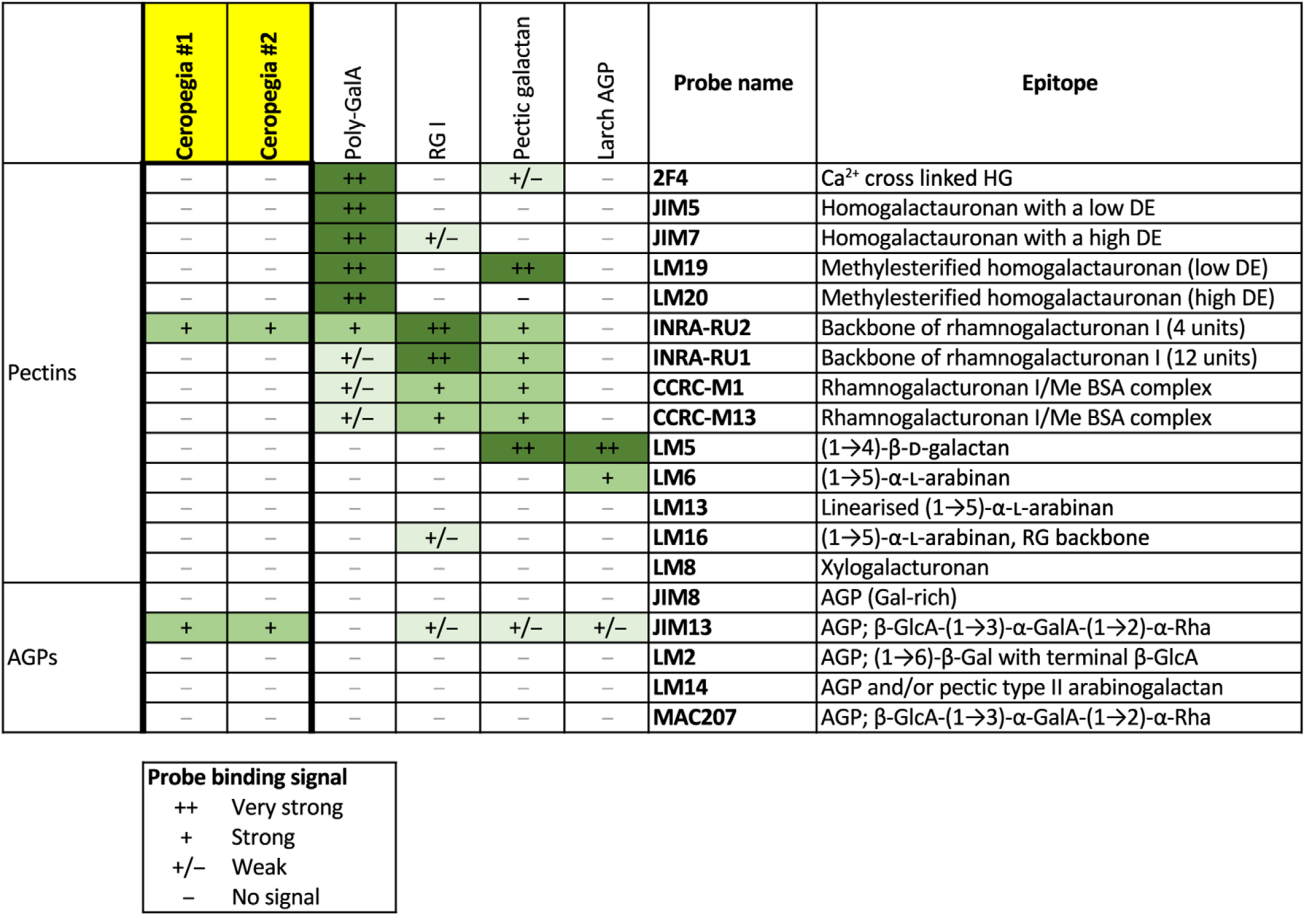
Binding of monoclonal antibodies to two samples of the *Ceropegia sandersonii* polymer. Signal strength is reported as very strong (++), strong (+), weak (+/−) or no signal (−).

Two samples of the *Ceropegia* polymer (10 μg each) washed off the gliding zone from several flowers were tested by comprehensive microarray polymer profiling. The composition of the hydrolyzed sample points to the presence of a pectic polymer or a glycoprotein, such as an arabinogalactan polymer. Forty‐six antibodies, related to pectins or arabinogalactan, were therefore chosen for our study. Only very few antibodies generated a signal, which appeared with the same relative signal intensity in both samples (Fig. 5). The strongest (still weak) signals were obtained with INRA‐RU2, binding to the RG‐I backbone of pectins (Nikiforova *et al*., 2022) and with Jim13, which binds to GlcA‐β1,3‐GalA‐α1,2‐Rha‐α in arabinogalactan proteins (AGPs) (Yates *et al*., 1996). The relatively weak signals suggest that the epitopes in *Ceropegia* are similar but distinct from the original epitope. These results also indicate that the published specificities need a cautious interpretation when it comes to structures prediction with GalA/GlcA. A full list with data for all antibodies is shown in Table S2.

### Chemical linkages of the *Ceropegia* polymer revealed by 2D NMR spectroscopy

**Fig. 6:**
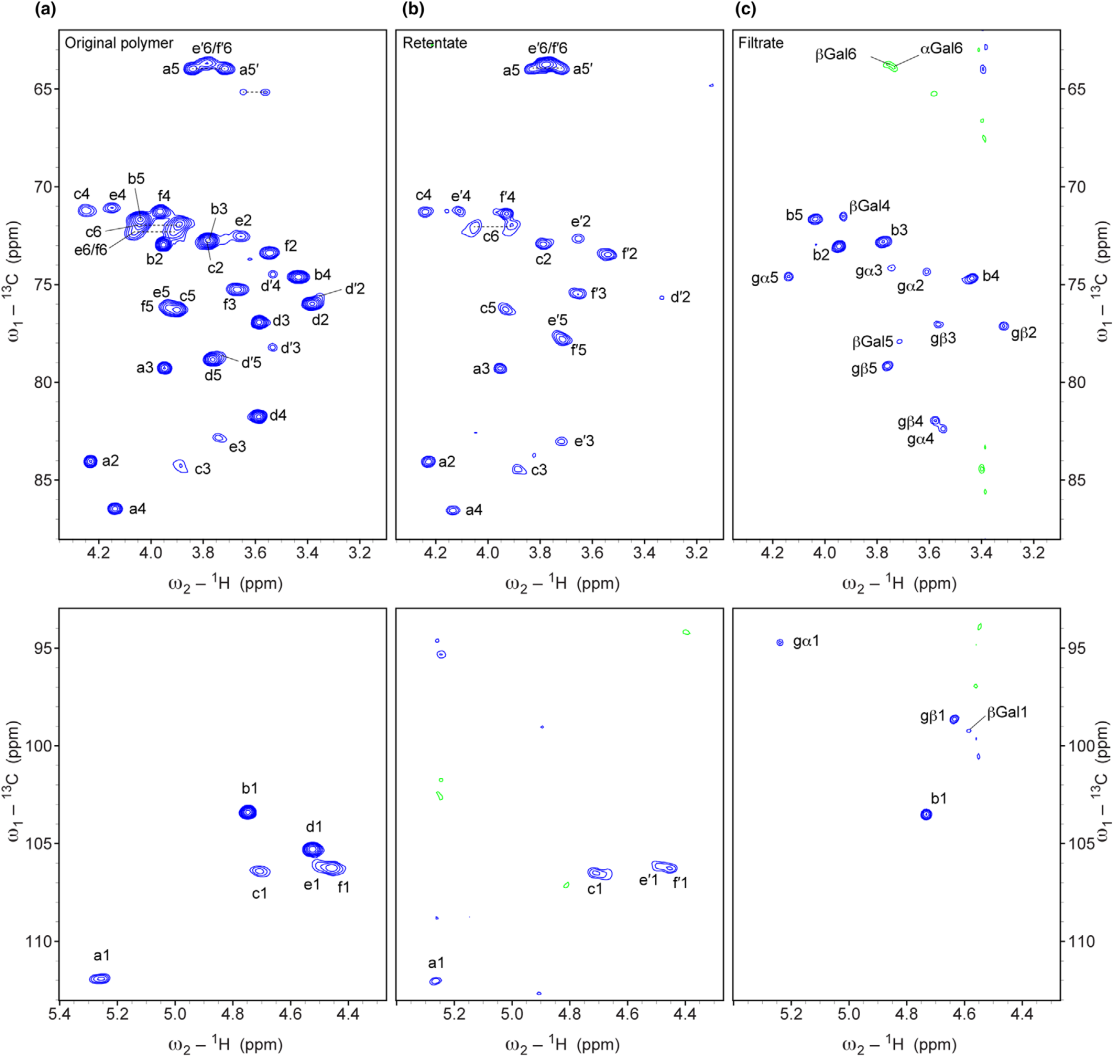
2D ^1^H–^13^C correlation spectra of the initial polysaccharide of *Ceropegia sandersonii* and of the products of enzymatic cleavage. (a) ^1^H–^13^C HSQC (heteronuclear single quantum correlation) spectrum of original polysaccharide with labels for each C–H pair. On bottom, the anomeric fingerprint region with all C1–H1 correlations is shown. The letter indicates the building block (named a–f in the order of the anomeric ^1^H chemical shifts from left to right); the number indicates the carbon position. (b) Comparable spectrum of the polysaccharide after cleavage with the enzyme FoBGlcA (Kondo *et al.*, 2021a). (c) Comparable spectrum of the filtrate after cleavage with the enzyme FoBGlcA.

The analysis of the samples by 2D NMR spectroscopy revealed six different building blocks/spin systems which were tentatively assigned to a, b, c, d, e and f, illustrated with two regions of a ^1^H–^13^C HSQC spectrum (Fig. 6a). This spectrum shows one signal for each C–H pair of all building blocks/spin systems. The signal height is indicated in these 2D contour plots by the number of contours. Each signal was assigned to the spin system (a, b, c, …) and the carbon number of the C–H correlation following state‐of‐the‐art 2D NMR analysis of carbohydrates (Fontana & Widmalm, 2023). Atom numbering is shown in detail in Fig. S3.

**Fig. 7:**
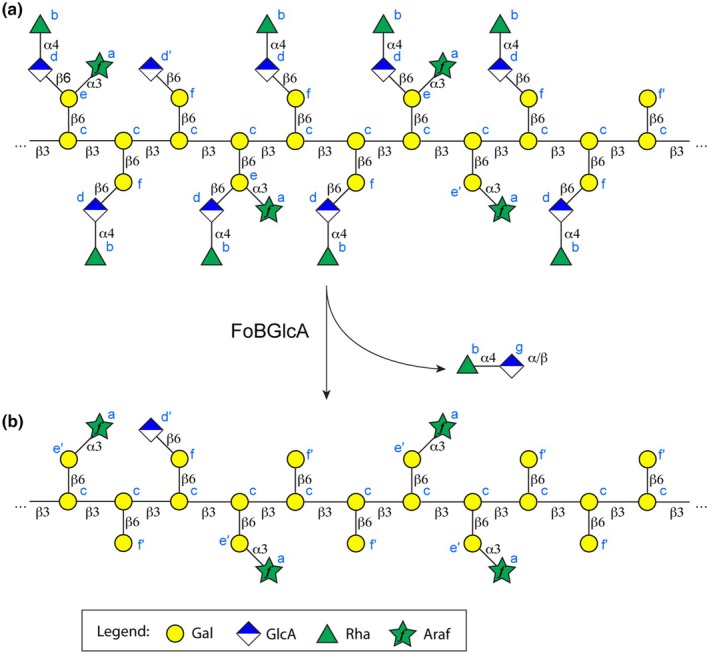
Structural model of the polysaccharide of *Ceropegia sandersonii* before (a) and after (b) enzymatic cleavage. The label of each unit corresponds to the assignment in the 2D nuclear magnetic resonance (NMR) spectra. The neighborhood of each side chain was tentatively chosen. Standard symbols of galactose (Gal), glucuronic acid (GlcA), rhamnose (Rha) and arabinofuranose (Araf) are shown together with the linkage type. The occurrence of each side chain corresponds approximately to the monosaccharide composition and the signal integrals in the polymer.

An analysis of these data resulted in the polymer model shown in Fig. 7. The full chemical structure is shown in Fig. S3. The sharp and strong signals of the spin systems b and d could be assigned to a Rha α1,4‐linked to a GlcA (Rha‐α1,4‐GlcA) side chain. Sharp signals of a polysaccharide are typical from flexible side chains, whereas the polymer backbone gives broader signals, as observed previously for other branched polysaccharides (Dobruchowska *et al*., 2008; Säwén *et al*., 2010). The medium strong signals of spin system a were assigned to terminal arabinofuranose units from side chains. The broad signals of component c could be assigned to a backbone of β1,3‐linked Gal repeating units. The spin systems e and f were assigned to Gal units that are β1,6‐linked to the (Gal‐β1,3‐)_*n*_ backbone. The components e and f distinguish themselves by further extensions: Spin system f is extended at O6 by a GlcA‐β or a Rha‐α1,4‐GlcAβ, and spin system e has in addition an Araf‐extension at O3.

The identity and the connections of the building blocks are supported by (1) chemical shift correlations across the glycosidic linkages (see Figs S4, S5), (2) matching chemical shift assignments of similar carbohydrates (see Fig. S2; Table S4) and (3) the prediction of chemical shifts of the proposed structural elements of the polymer with the web application CASPER (Lundborg & Widmalm, 2011) (see Table S4).

### Enzymatic cleavage of the polysaccharide to identify the unknown signal and to confirm the proposed structure of the polymer

Kondo *et al*. (2021b) recently characterized a glucuronidase from *F. oxysporum* that specifically cleaves the GlcA‐β1,6‐Gal bond of gum arabic. We incubated the *Ceropegia* polymer with the recombinant glucuronidase, which released a disaccharide. The disaccharide was separated from the polymer by a 10 kDa membrane. Upon hydrolysis, the sugars rhamnose and glucuronic acid appeared in equal molar amounts (Fig. 3c). In the HPLC analysis of the hydrolysis product of the remaining polymer, the unknown signal almost disappeared. We concluded that the unknown component was the disaccharide GlcA‐β1,6‐Gal, whose glycosidic linkage seems to largely resist acid hydrolysis. The chemical shift assignment matches perfectly to previously reported values for the same disaccharide (two‐dimensional NMR spectroscopy; Table S3) (Menestrina *et al*., 1998). This disaccharide was also observed after mild hydrolysis of Menestrina *et al*. (1998) confirming the resistance to hydrolysis.

To confirm the correctness of the NMR‐based polysaccharide structure independently, we also analyzed the enzymatically cleaved polysaccharide by 2D NMR spectroscopy. The ^1^H–^13^C correlation spectra of the remaining polysaccharide after treatment and the filtrate are shown in Fig. 6b,c, respectively. The spectra show that indeed Rha‐α1,4‐GlcA disaccharides are cleaved off: All signals of Rha (b) and GlcA (d) disappeared in the polymer spectrum, but appear in the filtrate spectrum. In the filtrate, we observe the same signals for α1,4‐linked Rha as in the initial polysaccharide, but the GlcA at the reducing end shows two sets of signals, namely of the α‐ and the β‐anomer.

**Fig. 8:**
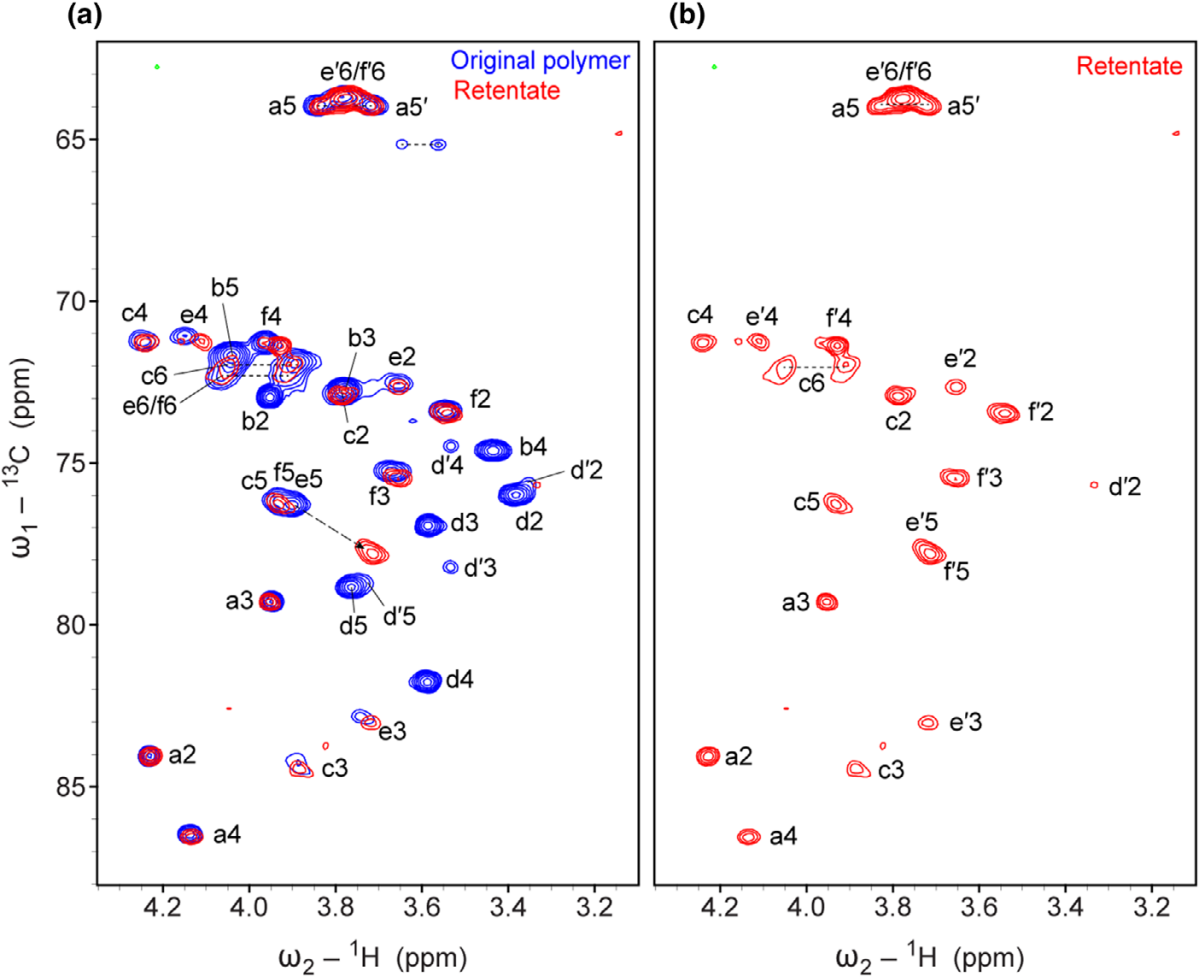
Comparison of the ^1^H–^13^C correlation spectra of the original polysaccharide of *Ceropegia sandersonii* and the glucuronidase‐treated polysaccharide. (a) Overlay of the ^1^H–^13^C heteronuclear single quantum correlation (HSQC) spectra of the original polymer in blue (with labels) and the polysaccharide after enzymatic cleavage in red. The largest differences are indicated by a dashed line. The letter indicates the building block (named a–f), the number the carbon position. (b) ^1^H–^13^C HSQC spectrum of the treated polysaccharide alone with labels.

Interestingly, the signals of the Gal residues where the Rha‐α1,4‐GlcA was previously attached to (spin systems e and f, after cleavage e′ and f′) showed marked differences in their C–H correlations (Fig. 8), especially C5–H5 and C4–H4, which are closest to the cleaved linkage, further confirming the model of the polymer.

The chemical shift assignments of the treated polysaccharide and the cleaved disaccharide were further confirmed by matching chemical shifts reported of similar entities (Fig. S2; Tables S5, S6). Using integrals of isolated signals in the ^1^H–^13^C HSQC spectrum allowed us to estimate ratios between different side chains, for example, between terminal GlcA and extended GlcA. The method is only semiquantitative because signals of moieties closer to the backbone have broader line widths and show smaller integrals. However, comparing, for example, isolated C3–H3 correlations of the different side chain Gal spin systems allowed the estimation that *c*. 32% of the side chains are branched, and 61% are linear, and only 7% terminate with Gal or Araf α1,3Gal; 93% of the side chains contain a GlcA and 77% a Rha. Of all GlcA‐containing side chains, 17% are not further extended by a Rha.

### The *Ceropegia* polymer is an arabinogalactan

The structure of the polymer shows similarity to the arabinogalactan from gum arabic (Cartmell *et al*., 2018; Kondo *et al*., 2021a,b). This includes the β1,3‐Gal backbone, β3,6‐Gal side chains with terminal Rha‐α1,4‐GlcA. To test whether the *Ceropegia* polymer is a novel AG, we used the Yariv‐precipitation and quantification method (Lamport, 2013).

**Fig. 9:**
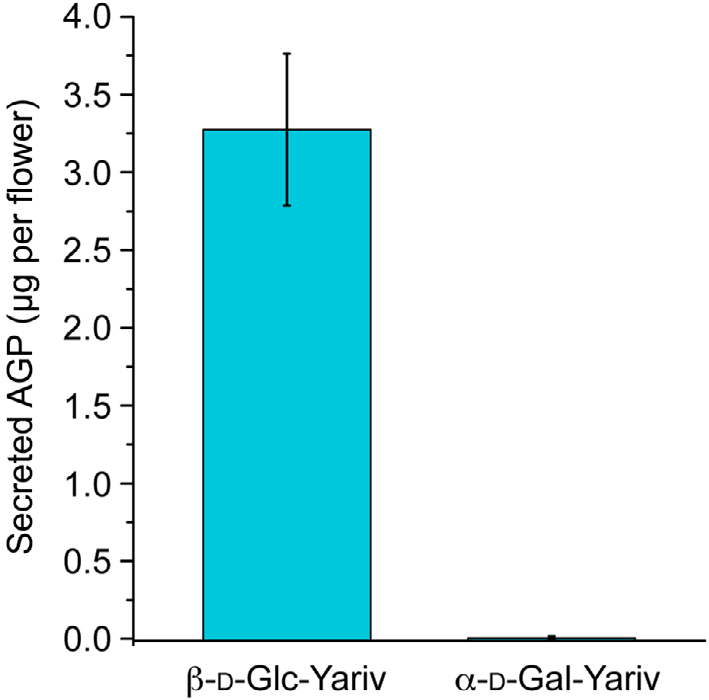
Quantitative determination of arabinogalactan (AG) (mean ± SD) in the *Ceropegia sandersonii* gliding zone wash offs. The samples from three biological replicates were incubated with β‐d‐glucose Yariv according to Lamport (2013). A red precipitate forms readily after the addition of the Yariv reagent. Incubation of the polymer with α‐d‐galactose Yariv, which is unable to precipitate AGs, served as a control.

Gum arabic was used as a reference AG. Using β‐d‐glucose Yariv, we obtained a strongly colored sample, whereas no signal was obtained with α‐d‐galactose Yariv, which serves as a negative control (Fig. 9). The calibration with gum arabic allowed us to estimate that each flower secretes *c*. 0.5 μg of polymer in the gliding zone.

Compared to other AGs, GlcA in *Ceropegia* is not methylated at the C4‐position (Silva *et al*., 2020) and the GlcA content in the *Ceropegia* polymer (*c*. 30%) is almost two times higher than in any other so far analyzed AGs. In general, the amount of GlcA in AGs varies from absent in larch (Ghosh *et al*., 2023) to 15–17% in Acacia Gum (Gum Arabic) (Goodrum *et al*., 2000; Aalbers *et al*., 2015). Polymers with GlcA are negatively charged, can retain water and prevent drying out, as is also true for other charged polysaccharides, such as the hygroscopic and viscoelastic polysaccharides with high surface tension in sundew droplets (Huang *et al*., 2015). In *Ceropegia*, the high content of GlcA likely explains the microscopic observation that droplets remain liquid in ESEM, whereas pure water evaporated under the same condition (Fig. 2). It also explains the rapid dissolving of the polymer in an aqueous environment, as observed by Vogel (1961).

As is true for the AG identified in the present study, some charged polysaccharides can switch between a liquid and a solid phases (Hoque *et al*., 2019). A diverse number of conditions might be a trigger for this phase transition including mechanics, magnetic or electric fields, change in ion or pH or hydrophobic effects (Gao *et al*., 2024). In the *Ceropegia*‐fly interaction, the contact between the legs/adhesive foodpads and the droplets could be such a trigger.

**Fig. 10:**
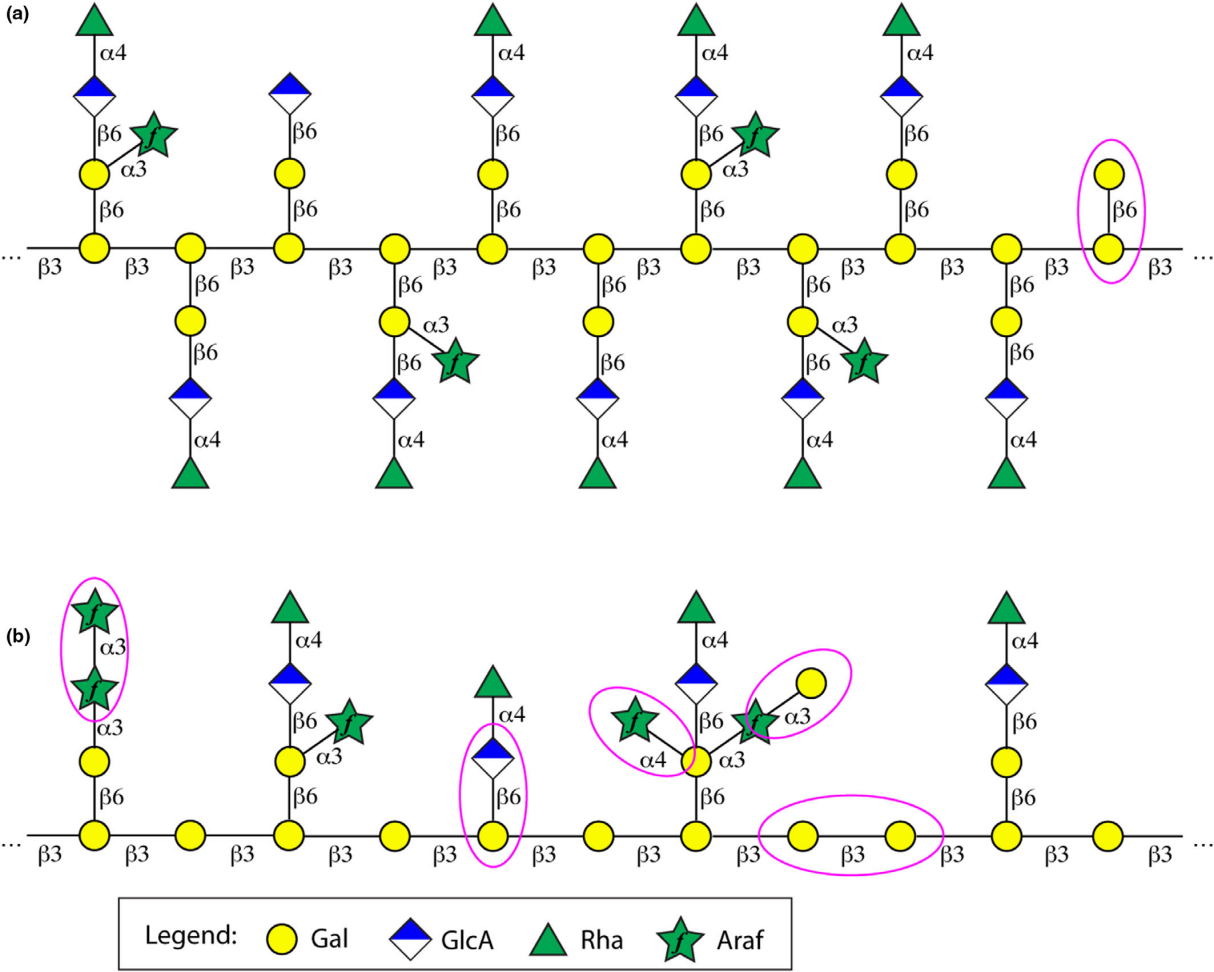
Comparison of the obtained structural model of the *Ceropegia sandersonii* polysaccharide with recent models of gum arabic. (a) Structural model of *C. sandersonii*. Standard symbols of galactose (Gal), glucuronic acid (GlcA), rhamnose (Rha) and arabinofuranose (Araf) are shown together with the linkage type. (b) Structural model of gum arabic based on models by Cartmell *et al*. (2018) and Kondo *et al*. (2021a,b). Differences are encircled in magenta.

The *Ceropegia* polymer is a type II AG with a β‐1,3‐backbone (Fig. 10a). The two side chains are rather short and contain only a single Gal attached to the backbone. The polysaccharide structure has close similarities with the polysaccharide of gum arabic (Cartmell *et al*., 2018; Kondo *et al*., 2021a,b) but also marked differences (Fig. 10b). Shared are the backbone of β1,3‐linked Gal repeating units, branches of β1,6‐linked Gal moieties, which in turn can be extended by Rha‐α1,4‐GlcA‐β1,6 and/or α1,3‐linked Araf. However, gum arabic displays a larger variety of side chains, including α1,4‐linked Araf and Gal‐α1,3‐Araf‐α1,3 extensions. Such side chains are absent in the *Ceropegia* polysaccharide, because NMR signals of Rha‐α1,4‐GlcA‐β1,6‐[Araf‐α1,4‐][Gal‐α1,3‐Araf‐α1,3‐]Gal‐β1,6 as reported previously (Cartmell *et al*., 2018) were absent. In addition, we do not observe Araf linked to the backbone as indicated in Cartmell *et al*. (2018) nor GlcA linked to the backbone as shown in the models of Kondo *et al*. (2021a,b). Neither do we observe Araf‐α1,3Araf linkages as shown in Cartmell *et al*. (2018).

Given the similarity of the polysaccharide of *Ceropegia* to gum arabic, our NMR study might be helpful for studying structural details of gum arabic. It might help to understand its synthesis and enzymatic degradation but also to pin down epitopes recognized by certain mAbs (Pattathil *et al*., 2010), whose exact recognition sites are not known yet.

### Is the arabinogalactan attached to a protein? To which?

Because AG are typically the carbohydrate part of arabinogalactan proteins (AGPs), we also looked for a (coupled) protein component. In NMR spectra of the droplets, no signals associated with typical protein resonances were detected. However, since the protein component in AGPs can be very small, the signal intensity might have been below the detection level. We then applied a typical proteomics approach using tryptic digests of the droplets and analyzed it with HPLC‐MS^2^ together with our transcript data of *C. sandersonii*. The top 20 proteins with a coverage of 1–25 peptides per sequence are listed in Fig. S6, among which three proteins contained C‐terminal extensions rich in predicted hydroxyprolines, which are typical for AGPs. Typically, AG are attached to hydroxyprolines (Hyp) and the conversion of Pro to Hyp depends on certain sequence motifs (Kieliszewski *et al*., 2011). All three protein candidates contained Pro‐Pro, Ala‐Pro or Ser‐Pro, which are typically hydroxylated and thus fulfilled these sequence requirements. The most abundant protein had a homolog in *Arabidopsis thaliana* (L.) Heynh. called AtPLAT2 (UniProt Q9SIE7), which only contains a Pro‐rich C‐terminus in *C. sandersonii* but not *A. thaliana*. The second protein also had a homolog in *A. thaliana* called AtPLAT1 (UniProt Q656660), which also lacked the Pro‐rich C‐terminal region. However, the second protein contained only one Pro‐Pro motif typical for hydroxylation of prolines. Interestingly, the third protein was a homolog of the *A. thaliana* protein Fasciclin‐like arabinogalactan protein (FLA1), which is an AGP, as the name implies. However, its abundance was only 6% compared to the PLAT2 homolog.

In conclusion, although we found three candidates for a putative protein component of the detected AG, we cannot tell whether they just coexist with the AG or are chemically bound to the AG as part of a larger AGP.

### Conclusions

We for the first time identified a polysaccharide from an anti‐adhesive surface of a plant. The droplets in the gliding zone of *C. sandersonii* flowers are composed of an arabinogalactan that contaminates the legs of the fly pollinators in the process of trapping them. Arabinogalactans are implicated to function in various plant growth and development processes, in plant microbe interactions and in abiotic stress responses (Silva *et al*., 2020). We add a new function to this compound class, showing that it is functionally even more diverse than previously thought. The physical properties of the identified arabinogalactan are well suited for temporally trapping the pollinators of *C. sandersonii*. Its negative charge prevents the liquid droplets from drying out so that they can easily contaminates the fly legs in liquid form, whereas the solidified droplets likely easily detach from the legs, allowing the flies to pollinate the flower, escape from the flower following pollination and export pollinaria to other flowers of *C. sandersonii*.

The corolla epidermis is responsible for the secretion of the droplets of arabinogalactan in *C. sandersonii*. These epiderms with their convex cells crowned by a bristle‐like central protuberance as production and release sites of secretions or scents of all kinds are the typical cellular equipment of the corolla upper surfaces in the tribe Ceropegieae (Vogel, 1961; Ehler, 1975). This anatomical prerequisite for the successful development of deceptive flowers in this species‐rich group of plants (the *Ceropegia* alliance encompasses over 700 species), with open (carrion) flowers or trap flowers, is probably as important as the much‐studied visual attractants and flower morphological specialties. Many open questions remain about the nature of the droplets which will require more work in the future. One curious fact that we were not able to explain is why the droplets are so stable on the flower surface, even under vacuum. However, when the droplets were touched, the droplet material flowed onto the contacting surface and then (almost instantly) solidified. This means that droplets are in an almost metastable state during flowering. This potentially could be due to fluid being continuously supplied from the plant beneath it. Alternatively, it could be possible that the polymer self‐organizes into a protective film on the surface keeping the droplet stable. These strange material characteristics, the small droplet size and the limited time period with flowers available made physical characterization of the droplet material very difficult. Attempts were also made to visualize the 3D microstructure of the plant cells below and within the droplet using Cryo‐SEM. No clear channel was observed, and no droplet skin was seen. As such, this work was focused on understanding the chemical composition of the droplets. Future studies that perform quantitative attachment/traction force tests and record more physical data on the droplets and their interaction with the fly legs will help to elaborate more on the quantitative functioning of these droplets.

## Competing interests

None declared.

## Author contributions

PF, JWCD, ME, MS, RT and SD planned and designed the research. PF, MS, CM, CR, PB, KH and ME performed experiments. PF, MS, CR, PL, KH and RT analyzed data. PF, MS, SD and RT wrote the manuscript, with some parts written by ME and UM. All authors contributed to the manuscript. PF and MS contributed equally to this work as first authors.

## Disclaimer

The New Phytologist Foundation remains neutral with regard to jurisdictional claims in maps and in any institutional affiliations.

## Supporting information


**Fig. S1** Proton 1D spectra of the polysaccharide hydrolysate in comparison with spectra of reference monosaccharides.
**Fig. S2** Symbol presentation of oligo‐ and polysaccharides used as a reference for comparison of NMR data.
**Fig. S3** Chemical structures of the *Ceropegia sandersonii* polysaccharide and the released glycan.
**Fig. S4** Observed NOE cross‐peaks connecting the building blocks.
**Fig. S5** Observed long‐range ^1^H–^13^C correlations connecting the building blocks.
**Fig. S6** Sequences of the most abundant proteins identified by proteomics of the *Ceropegia sandersonii* droplet solution.
**Methods S1** Efforts to identify the protein component of the *Ceropegia* polymer by LC‐HRMS.
**Table S1** Applied gradients used for HPAEC‐PAD analysis.
**Table S2** Binding of monoclonal antibodies to two samples of the *Ceropegia sandersonii* polysaccharide as detailed in the table.
**Table S3** Observed chemical shifts of the disaccharide GlcAβ1,6Gal.
**Table S4** Observed chemical shifts of the intact polysaccharide of *Ceropegia sandersonii*.
**Table S5** Observed chemical shifts of the polysaccharide of *Ceropegia sandersonii* after enzymatic cleavage.
**Table S6** Observed chemical shifts of the cleaved disaccharide Rhaα1,4GlcA.
Please note: Wiley is not responsible for the content or functionality of any Supporting Information supplied by the authors. Any queries (other than missing material) should be directed to the *New Phytologist* Central Office.

## Data Availability

The authors confirm that the data supporting the findings of this study are available within the article and its Supporting Information (NMR chemical shifts in Tables S3–S6). The mass spectrometry proteomics data have been deposited to the ProteomeXchange Consortium via the PRIDE (Perez‐Riverol *et al*., 2025) partner repository with the dataset identifier PXD062347: https://www.ebi.ac.uk/pride/archive/projects/PXD062347.
