# Supplementary material for: Deceptive Ceropegia sandersonii uses an arabinogalactan for trapping its fly pollinators
Source: New Phytol. 2025 Apr 20;246(6):2738–52. doi: 10.1111/nph.70144 (PMC12095969; doi:10.1111/nph.70144)
Supplement: Supplementary file 1 — Fig. S1 Proton 1D spectra of the polysaccharide hydrolysate in comparison with spectra of reference monosaccharides. Fig. S2 Symbol presentation of oligo‐ and polysaccharides used as a reference for comparison of NMR data. Fig. S3 Chemical structures of the Ceropegia sandersonii polysaccharide and the released glycan. Fig. S4 Observed NOE cross‐peaks connecting the building blocks. Fig. S5 Observed long‐range 1H–13C correlations connecting the building blocks. Fig. S6 Sequences of the most abundant proteins identified by proteomics of the Ceropegia sandersonii droplet solution. Methods S1 Efforts to identify the protein component of the Ceropegia polymer by LC‐HRMS. Table S1 Applied gradients used for HPAEC‐PAD analysis. Table S2 Binding of monoclonal antibodies to two samples of the Ceropegia sandersonii polysaccharide as detailed in the table. Table S3 Observed chemical shifts of the disaccharide GlcAβ1,6Gal. Table S4 Observed chemical shifts of the intact polysaccharide of Ceropegia sandersonii. Table S5 Observed chemical shifts of the polysaccharide of Ceropegia sandersonii after enzymatic cleavage. Table S6 Observed chemical shifts of the cleaved disaccharide Rhaα1,4GlcA. Please note: Wiley is not responsible for the content or functionality of any Supporting Information supplied by the authors. Any queries (other than missing material) should be directed to the New Phytologist Central Office. [file NPH-246-2738-s001.pdf]

New Phytologist

New Phytologist Supporting Information

Article title: Deceptive *Ceropegia sandersonii* uses an arabinogalactan for trapping its fly pollinators
Authors: Philipp Feichtlbauer, Mario Schubert, Caroline Mortier, Christof Regl, Peter Lackner, Peter Briza, Klaus Herburger, Ulrich Meve, John Dunlop, Michaela Eder, Stefan Dötterl, Raimund Tenhaken
Article acceptance date: 26 March 2025

The following Supporting Information is available for this article:

Supplementary Figures

Supplementary Tables

Supplementary Methods

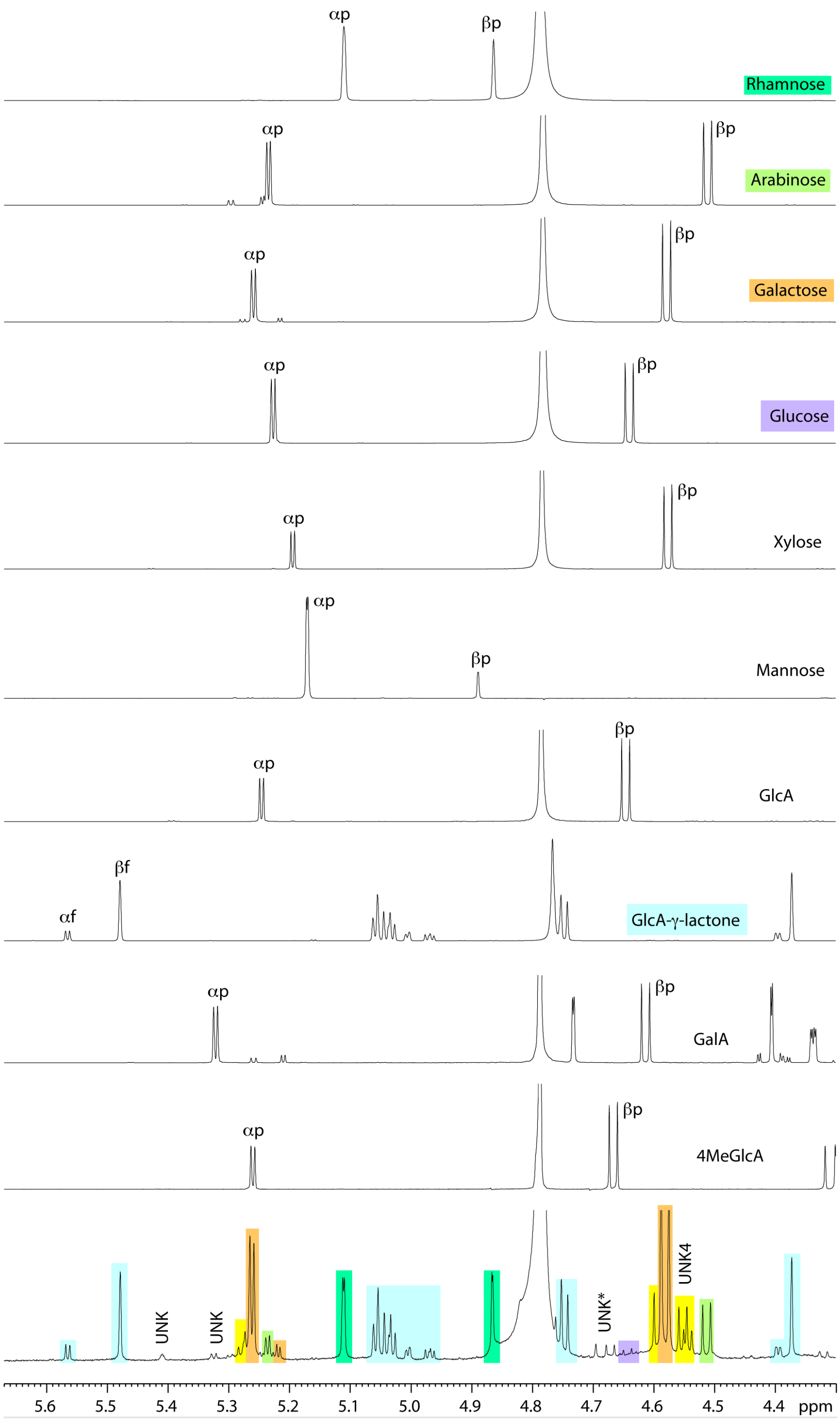
αp
βp
Rhamnose
αp
βp
Arabinose
αp
βp
Galactose
αp
βp
Glucose
αp
βp
Xylose
αp
βp
Mannose
βp
αp
GlcA
βf
αf
GlcA-γ-lactone
αp
βp
GalA
αp
βp
4MeGlcA
UNK
UNK
UNK*
UNK4
5.6
5.5
5.4
5.3
5.2
5.1
5.0
4.9
4.8
4.7
4.6
4.5
4.4
ppm

Fig. S1 ^{1}H 1D spectra of the hydrolysate (bottom) of the *Ceropegia sandersonii* droplets in comparison with spectra of reference monosaccharides. Matching signals of monosaccharides are color-coded.

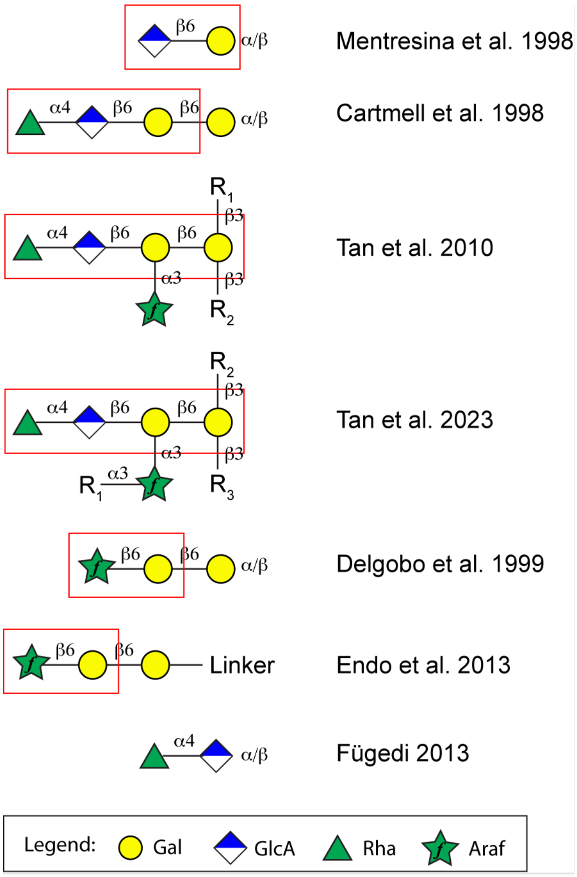

Fig. S2 Symbol presentation of oligo- and polysaccharides used as reference for comparison of NMR data. The building blocks of relevance are highlighted by a red box. (Fugedi, 1987; Menestrina *et al.*, 1998; Delgobo *et al.*, 1999; Tan *et al.*, 2010; Endo *et al.*, 2013; Cartmell *et al.*, 2018; Tan *et al.*, 2023)

Backbone:

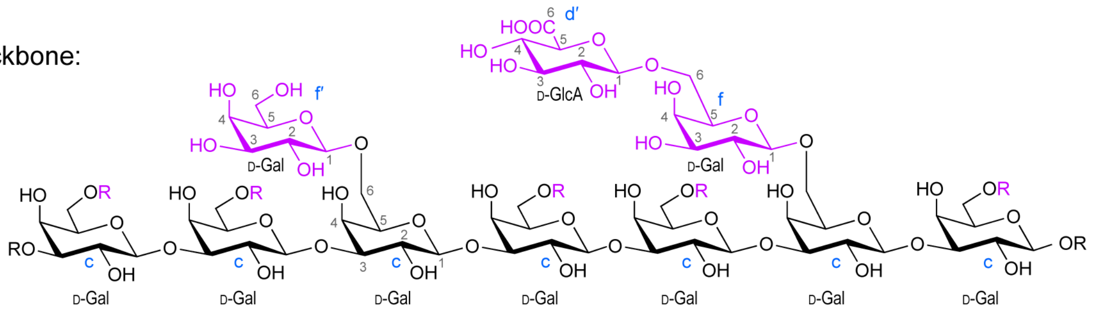

Other observed side chains:

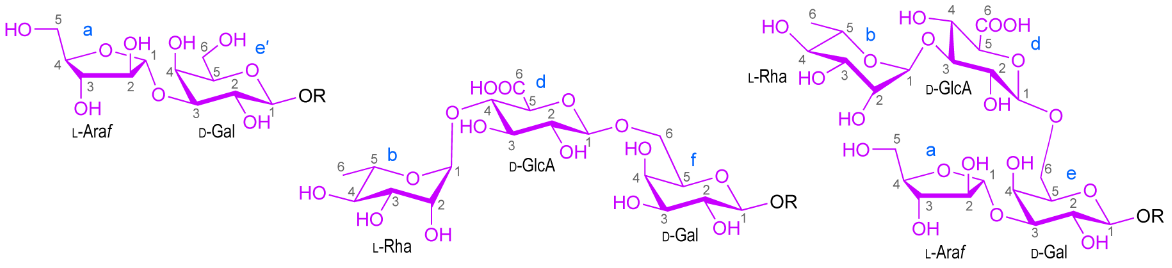

Enzymatically released glycan:

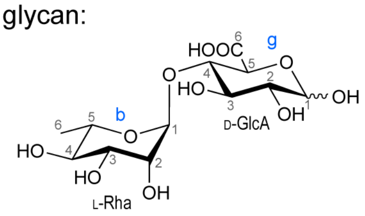

Fig. S3 Chemical structures of the *Ceropegia sandersonii* polysaccharide and the released glycan. The side chains are shown in magenta, their order is tentatively. The different building blocks are marked with letters a to g corresponding to the observed NMR spin systems.

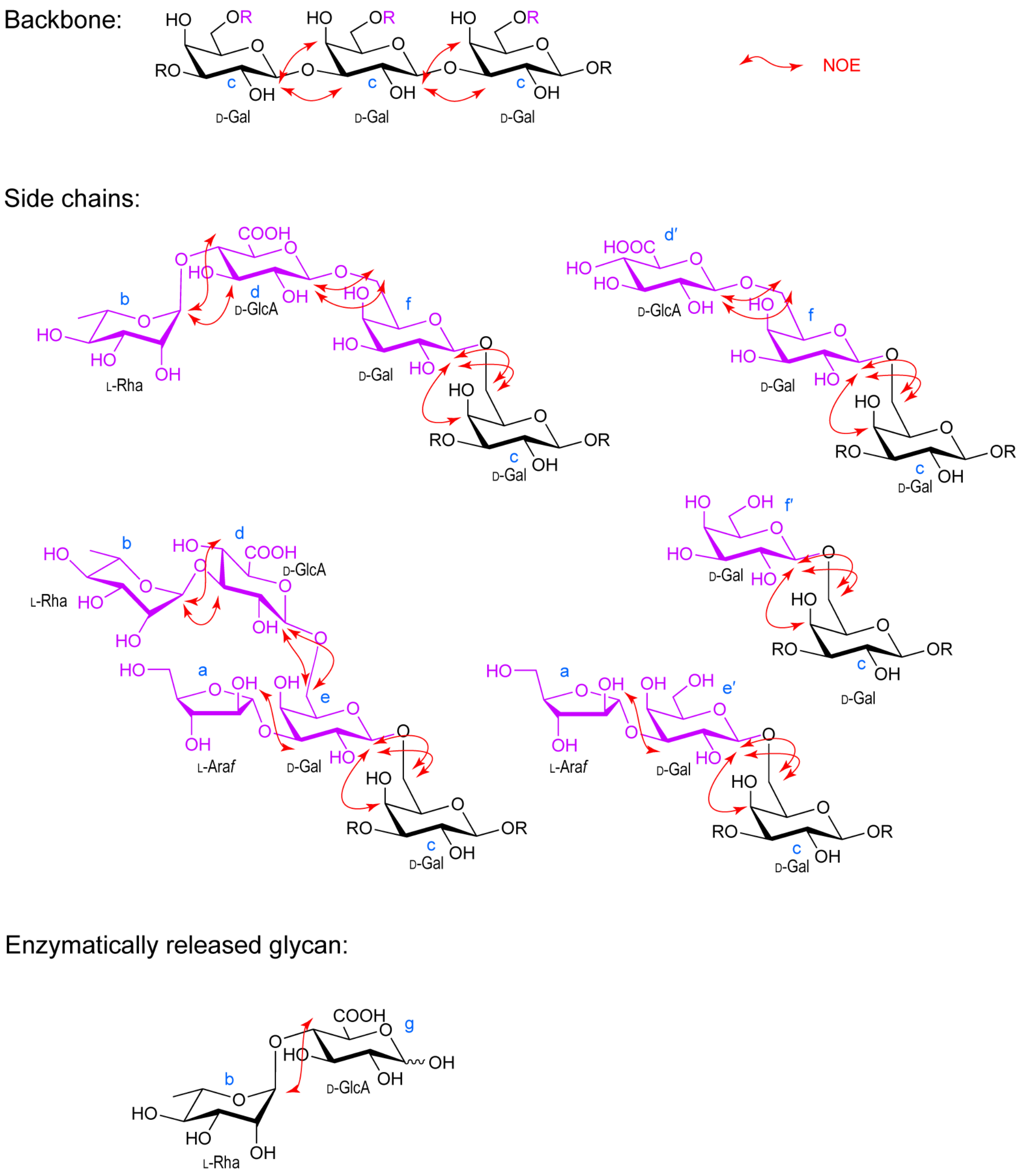

Fig. S4 Observed NOE cross peaks connecting the building blocks indicated with red arrows on the chemical structure of the *Ceropegia sandersonii* polysaccharide and the released glycan. The side chains are shown in magenta, their order is tentatively. The different building blocks are marked with letters a to g corresponding to the observed NMR spin systems.

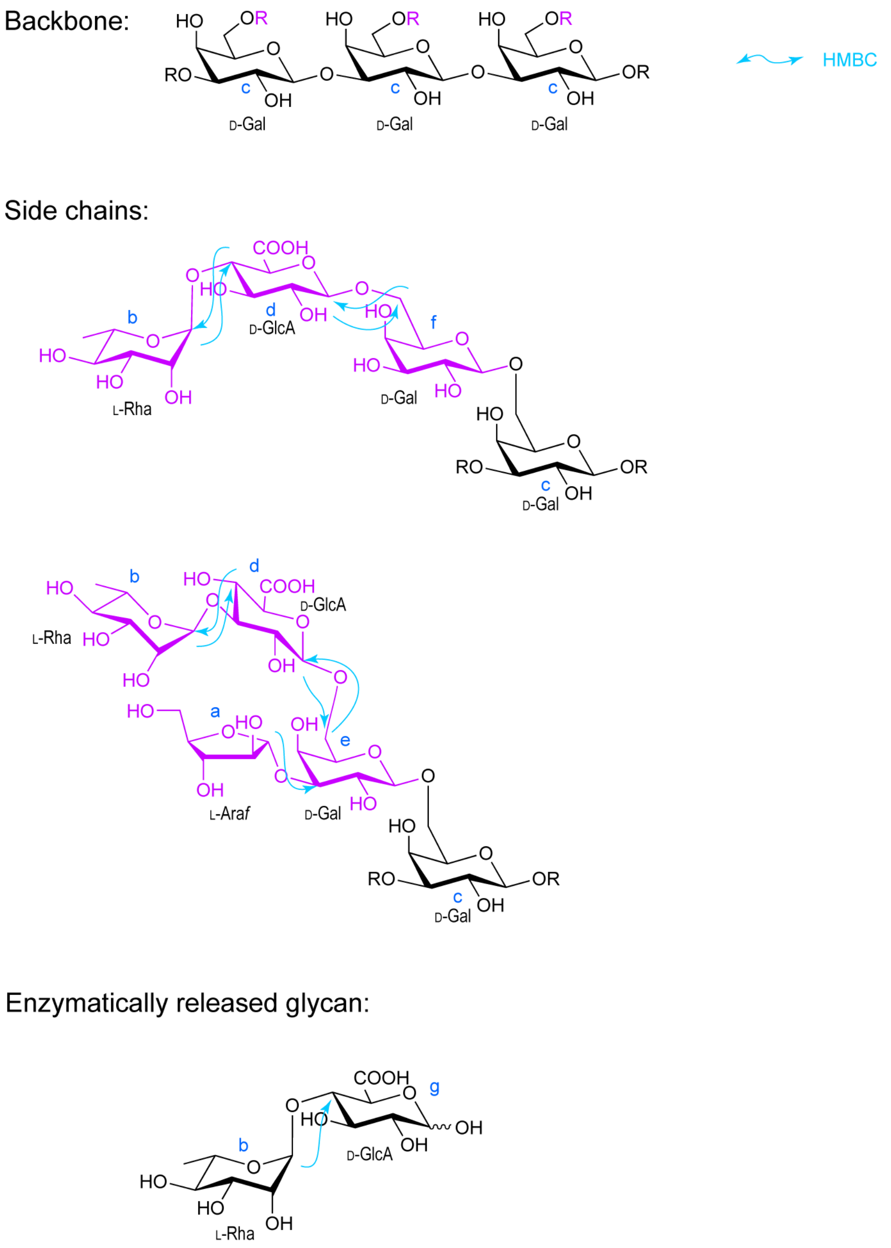

Fig. S5 Observed long range ^{1}H-^{13}C correlations connecting the building blocks indicated with cyan arrows on the chemical structure of the *Ceropegia sandersonii* polysaccharide and the released glycan. The side chains are shown in magenta, their order is tentatively. The different building blocks are marked with letters a to g corresponding to the observed NMR spin systems

TRINITY_DN520_c0_g2_i1 4 (100% relative abundance, 10 peptides, 10 unique peptides)
homolog to AtPLAT2 (Q9SIE7), identities: 55/160 (34%)
IRFSSFLLLL LATFTPSIVA QTSVEASQTL DGAKICMYTL YVKTGDVQNA GTNSTIR**MDF**
TDSTGWTIRI SNIAAIGLMG AGHNYFQRGQ VDIFGFGIGC MRTPICRVAI SSDGTGKNPA
WFLDTIQISY TGIGRRCHET VFGIYRWISD SNGLNFVLDN CGIPPFPPVR PFPPFPPAPF
PYHVKNEKVK ELVAEKIMEE EEVAPTSVEV ASVVLKPRMS FTEEN

TRINITY_DN1987_c0_g1_i2 2 (81% relative abundance, 7 peptides, 7 unique peptides)
homolog to LTP2 (Q43194), identities: 52/98 (53%)
NNMQRSPLPI STNKYNFNLQ SIYNTKNPIL TMESVRKMAG AAVIMIIINS VCMMMFSSSE
AAITCSQVNS NIGPCIKYIV NKAILNPSDS CCKGVKTLNS AATSTPDLRI ACKCLKDIAR
KFPGIDMKKA GAIPGKCGVK **LPFQISLSTD CNK**VQ

TRINITY_DN6779_c0_g2_i1 6 (63% relative abundance, 11 peptides, 11 unique peptides)
homolog of endochitinase (P85084), identities: 137/244 (56%)
EIRNMIMKYY YMILIIILSC FFTAFSSSSS ILADDQDYSN NNITISSVIN STIFNQMLGH
SMDSRCPSNG FYTYDAFISA ATSFPAFGTT GDNDTQKREI AAFLGQTSHE TTGGWPNAPD
GPYAWGYCII IKNKIQNHTF CNSRRVKCPP GKSYYMR**GPI QLIGSDNYYR** VGEEIGEDLL
NNPDLVETNA TISFKTAIWF WMTDYFSTPS CHDVIVGKWK PSADDKRYGR LPGYGTITNI
FNGESECGRG GVITPAENDR IQFYKRYCNM FGIYCGGPLD CRYQDPFGIR ILN

TRINITY_DN1915_c0_g1_i8 6 (62% relative abundance, 18 peptides, 18 unique peptides)
homolog of aspartyl protease family protein (Q8S9J6), identities: 238/424 (56%)
IGPGRFGEGD LNSYSSSSFL IMANSNNYFF FFFNFLHPIS LLVFLLSFSN LKGGGCAMLE
KTHEKVIAEA HYHTVHVSSL LHIQSSCKTS SPHKGHNTKS ASLEVIHKHS ACKKDTIKEP
TLVEILAADE SRVNSIHGRR FLNSGLINNK YRNSKANLPA KPGVTIGSGN YIVNVGLGTP
ARTLTLVFDT GSDLTWTQCQ PCARSCYDQQ DPIFDPSKST SYSNITCNST QCSALPSATG
NKPGCIASTS TCVYGIQYGD QSFSVGYFSK DKITLTQVDS FDNFLFGCGQ NNQGLFGKTA
GLLGLGRDPL SIVSQTSQKY GKFFSYCLPN KSGGSKGHLT FGKKNLPTNI VYTPLKNTNQ
GTFYFVDIIG INVGGQLLSI NQSIFKNAGT IVDSGTVITR **LPQTGYDALK** TAVKKQMSQY
PTAASVSILD TCFDLSNYTS VTVPKLVFIF GGNSKVELPV LNILVAAKQS ILCLAFAGNG
DDSSVGIYGN IQQQTFEVVY DVAGGKLGFG PSGCS

TRINITY_DN520_c0_g1_i3 5 (40% relative abundance, 5 peptides, 5 unique peptides)
homolog to AtPLAT2 (Q9SIE7), identities: 60/164 (37%)
REAKKREKRK KMAIIRFSSF LLLLLATFTT SIVAQNSVAA LKVDGAKICL YTIYVK**TGDI**
EMAGTDSKIS MNFTDYTGSK LIVENLTSIG LMGEGHDYFK RGKVDIFGVG TQCLLTPICS
VEVALSSNGN GILPDWYLET IQISFTGIDG GCRQTVFIIN EWLTTSDILS YEANECSIRP
FPSPFHGSIN EEGHEELIVA KKLMLKEKVA QPSVVAAAGE LKPLRSVNHN SFSEQ

TRINITY_DN4435_c33_g1_i1 4 (38% relative abundance, 1 peptide, 1 unique peptide)
homolog to LPT2 (P82353), identities: 29/57 (51%)
ITAFQSGAPQ SSGCCVSLKS QQPCLFQYAK NPALR**QYITN PNAK**KVAQSC MVPLPKC

Fig. S6 Sequences of most abundant proteins identified by proteomics using all potential reading frames of the transcriptome of *C. sandersonii*. Prolines in sequences predicted to be hydroxylated are indicated in magenta, otherwise grey. Clusters of such hydroxyprolines are typically found in arabinogalactan proteins, typically in unstructured regions or tails. Sequence regions covered by a peptide found in the MS analysis are underlined. The most abundant peptide is marked in bold letters. The corresponding relative abundance as well as the number of matched peptides and uniquely matched peptides is denoted in the parentheses after the Trinity accession code.

TRINITY_DN3751_c6_g1_i3 5 (36% relative abundance, 6 peptides, 6 unique peptides)
homolog to LTP (Q39950), identities: 73/122 (60%)
KKKRKKKKKE KKIKKMEMMG KKFVLFMVVA IMVALIKPGV EATITCGQVS GSLAPCIPYL
TKNAPMGECC DGVQKLNSQA QTTPDRQTAC GCLKSAYK**SM TGINPALAAG LPAK**CGVSIP
YKISPDTDCT KVR

TRINITY_DN520_c0_g1_i18 5 (35% relative abundance, 7 peptides, 0 unique peptides)
homolog to AtPLAT1 (Q65660), identities: 54/130 (42%)
KEKMAIIHFS SLLLLLLATF TTSIVAQTSV EAPKVDGAKI CLYSVYVKTG DIEKAGTDSK
ISMNFTDYTG RTLVVQNLR**S IGLMGNDHDY FER**GQVDLFS TGSHCVRTPI CSVSILSDGT
GILPAWYLET IQISFTGIGR NCYKTVFNIN KWLSSSGSLS YVANNCRFRP FPPFPFSFLG
SNEEEQELAA KKIMLEEEVA KPSVVAAGEL KPLRSVNHNS FSEQ

TRINITY_DN827_c3_g1_i1 1 (30% relative abundance, 1 peptide, 1 unique peptide)
homolog to LTP (Q43681), identities: 37/80 (46%)
LVMMIIIVLL AGDEVKVSKE QNCNPTELSS CLPAFQYGAP TSSGCCGCLK **SQQPCLCQYA**
KHPGFRQFIT NPNANIVAQS CKVPFPKC

TRINITY_DN1483_c0_g1_i3 6 (26% relative abundance, 1 peptide, 1 unique peptide)
homolog to E3 ubiquitin ligase UPL6 (Q8RWB8), identities: 489/691 (71%)
FFHGTSGLHQ AVDFATVATP LLGALPSITP SNEGNKQNGI VEDDEMVYGD EHGEEVLHGD
IQLQIYNAID SRFLLQLTNV LLGGVSLPDK SCNGVPSSKD VAAVGAACAF LHVTFNILPL
ERIMTVLAYR TGLVPVLWNF MKRCHVNNIW SSLSRQSAYL PEGAPGWLLP LAVFCPVYKH
MLMIVDNEEF YDQEKPLPLD DIRFLIVILK QALWQLMWLN PVVPPNFSKS SSSINAMKQQ
PLEFLQHKVC VTTSELLSQL QDWNNRRQFT PPSDFHADGV DENFISQAWT ENTKANATLK
LAPFLLPFTS RAKIFHSQLA AVKERHAHHD AFSRIRFRVR RDHILEDAFS QLNALSEEDI
RGQIRITFIN EFGVEEAGID GGGIFKDFME SVTRAAFDVQ YGLFKETADH LLYPNPGSGL
VHEMHHQLFY FLGTVLAKAM FEGILVDIPF ATFFLSKLKQ KYNYLNDLPS LDPELYRHLI
FLKHYEGDIS DLELYFVIVN NEYGEQTEEE LLPGGKNVRV TNENVITFIH LVANHRLNAQ
IRQQSSHFSR GFQQLIQKEW IDVFNEHELQ LLISGSVDGF DIDDLRANAN YGGGYSAEHY
VIQMFWEVVK HLSLENQRKF LKFVTGCSRG PLLGFKNLEP LFCIQRAAGR ASEEALDRLP
TAATCMNLLK **LPPYKSKEQM EQK**LLYAINA AAGFDLS

TRINITY_DN3376_c1_g1_i2 3 (25% relative abundance, 8 peptides, 8 unique peptides)
homolog to pathogen related protein 1 (P11670), identities: 100/159 (63%)
KKKKKKKKKK KKKKEIMGST RLMAAVMITI TTTLFSLTWL PSGATAQNSN QDYLNAHNAA
RAQVGVGPMI WDNQLAAYAL NYANSQKGRC PNLSHSSGPY GENLAAGTGD FTGR**QAVNLW**
VNEKQFYDYG SNSCAAGRVC GHYTQVVWRN SVRLGCARVR CSNNTWWYVI CSYDPRGNYV
GQRPY

TRINITY_DN7514_c0_g1_i1 3 (25% relative abundance, 25 peptides, 25 unique peptides)
homolog to subtilisin-like protease (Q65351), identities: 543/761 (71%)
SVQTIASSLS LSLSLSLYIY IYITAEHFQN ISPTPFPSFS FTSLGLNMGN TNFHGALIAL
VLLLFVCHAS SQAAMLKKNT YIVHMAKSEM PESFDDHTSW YDSSLKSVSD SSQMIYTYND
VIHGFAARLT PEEARLLRKR SGIVSVLPEM RYELHTTRTP SFLGLERSAS LFPESDSASD
VVIGVLDTGV WPESKSYDDT GMGPIPASWK GVCQTGTKFT TANCNRKLIG ARYFSDGYEA
TLGPIDVSKE SKSPRDDDGH GTHTSSTAGG SLVTGASLFG YANGTARGMA PHARLAIYKV
CWIGGCFSSD ILMALEMAIQ DNVNILSLSL GGGMSDYYRD SVAIGAFAAM QKGILVSCSA
GNAGPSPYSL SNVAPWITTV GAGTLDRDFP AYVSLGNGKK FAGVSLYRGP DLTTKMFPFI
YAANASNVTN GNLCMTGTLI PEKVGKIVL CDRGVNARVQ KGSVVK**AAGG AGMVLSNTAA**
NGEELVADAH LLPATAVGEK SGDAIKDYLI ADANPTVTIL FEGTKVGIEP SPVVAAFSSR
GPNSITPQIL KPDVIAPGVN IIAAWTGAVG PTGLAEDGRR VGFNIISGTS MSCPHVSGLA
ALLKGAHPTW SPAAIRSALM TTAYSAYKNG KALEDLATGQ SSTPFDLGAG HVDPVSALNP
GLVYDLEATD YLNFLCALNF TSLQIYSLAK SNFSCDPGKK YSIGDLNYPS FAVLLETQTE
GGGNGGSKTG SSVVKHSRTL TNVGAPATYK VTTTVDDPSV KISVVPETLT FTAMNEKKSY
TVTFSTSSKP ANTNVFGRIE WSDGKQHVVG SPVAVSWT

Fig. S6 continued.

TRINITY_DN1706_c0_g2_i1 5 (14.1% relative abundance, 8 peptides, 8 unique peptides)
homolog to AtPLAT2 (Q9SIE7), identities: 66/171 (39%)
LIIQKKMSER TRFFAYLLLF LTLSSVAVTA HKSDCAYTIV IKTADEAKAG TDAKIKITLG
DFKGKSVHVV DLEK**WGIMGS DYDYYER**GST DLFSGKDNCL RQPVCRLKLE SNAKGESPGW
KIDYVDVTTV IPEVGCKQAK FKVDQWLGVE GKNELSIVHE ECIDYLKNNK KKQQQSTTRS
LVKGKSSNHN MV

TRINITY_DN8840_c0_g1_i1 1 (12.2% relative abundance, 6 peptides, 6 unique peptides)
homolog to AtPLAT1 (Q65660), identities: 60/140 (43%)
LIKEMENYRC FSLLVLLLLI AFSSATTQAY VHKCVYTFFV KTGDILSAGT DSTIGLTLSD
AGGGSIDIPD LKKWGIMEWR HDYFERGNLD IFSGR**DTCLE PPICNLK**LVS NGVGTHPAWN
VINVFVGISR PDGPCTEINF PVNGWLGAFG HPFVLTVNLP GCPKRSTLKN AVESSTKALD
Y

TRINITY_DN791_c0_g1_i2 3 (11% relative abundance, 4 peptides, 4 unique peptides)
homolog to Sneakin-2 (Q93X17), identities: 56/107 (52%)
LNPTQPNPTC HHHHYYFLCL YNKDSVLIIS FISLNNKYKQ TMAFLKAAIA ALFLCLLFLH
STIALQGVNI PSSSSSPSPA PLPQIKK**IDC GSACGVR**CSK TKRPNLCKRA CGSCCVKCNC
VPEGTYGHYE TCPCYFNLTT HNNTRKCP

TRINITY_DN2808_c0_g1_i1 2 (11% relative abundance, 11 peptides, 5 unique peptides)
homolog to Ervatimin-C (A8DS38), identities: 159/365 (44%)
RTELTKMNSS SNLFIIIIFL SISQTINSIN NNGESVSWQR TDNELMALFE EWMMQHQKFY
SSSSSVLGEK IDRFEIFKEN LRYIDEHNNL PNTTFQLGLN QFSDLTYEEF ESMYLSRISM
NKMSPINQEE LELESDDNYG GDQFNYSTLT LPNYVDWRKE GAVNPIKNQG YCGSCWSFTT
VASVEAINKI KNGKLVSLSE QMLLDCVTSN GGCNGGTAAN SFNYMKTYGV ALNEDYPYVG
FKGVCQNKPI AVKIDGFNLV LPRLELK**LLP AVANQVLTIA IK**AGSEDFRH YKSGVFSGQC
GNKFSHGMNV VGYSFKNGKL YWILRNSWGE TWGEKGYMRI AGLVSGYERG YCGIAEEATY
PIMSTSVNQE LLSVV

TRINITY_DN4498_c1_g1_i2 1 (10% relative abundance, 5 peptides, 5 unique peptides)
homolog to PsLTP1 (A0A161AT60), identities: 61/113 (54%)
TMYTFKNMMI MIMSVMMVLC MVMFSSPYSG AEAAVTCGLV SSSVAPCMSY LQNK**AATIPS
TGCCSGIK**SL SSAASSTPDR QAACKCLKVL AGQIKGIDMN KAAGLPGKCS VNVPFKISLS
TNCDTVR

TRINITY_DN3849_c2_g1_i2 2 (7% relative abundance, 8 peptides, 6 unique peptides)
homolog to AtPLAT2 (Q9SIE7), identities: 61/184 (33%)
RAKMVRSFFS TLLLLFTLLV ATVVAHKEAI SSLSVDRQCA YSLFVRTGDR LNSGTNAKIN
VTLVDSHGES FVIHNLVKFG LMK**TGHNYLE NGNTDFFSVK** GKCLTNPICK LILKSDGSGY
RPSWFVVYLK VVQNEANVPC FESTFNIGKW IGSDYHNDGG YGVVKGGGIK HAPFELVVTL
DECVKRARHS KRIDHSH

TRINITY_DN11309_c0_g2_i3 4 (7% relative abundance, 18 peptides, 17 unique peptides)
homolog to isoflavonoid 7-O-beta-apiosyl-glycosidase (A3RF67), identities: 230/469 (49%)
FGFGSSAYQT EGAWNVDGKG PSQWDNFTHT YPERIEDKSN GDNATNAYYL YQEDIRQLEI
MNADAYWFSI SWSRVLPNGR INGGRGINYK GIEYYNNLID VLISKGLKPC VTMYFFDLPQ
ALQDEYGGLL NGPKFREDFV QYADLLFRTF GDRVKQWVTI NDPLRIVTLA YDLGVFPPNR
CSSWVNKDCF GGDSATEPYI VAHNLLLAHA DVVQLYRSEY KEKQEGIIGI ILQGTWPRPY
NYSNLADHKA AGRYIDFTVG LFLSPITFGY YPQTVRDYVG ER**LPEFTEEQ SLLLR**GSIDF
LGFNYFSARF VYELNIPREP RSYFDDIHVG VNYTNVNGTL VGEKLGVSWQ YSYPKGIWRY
LRYIKRRYNS PIIYITGNGA SEVNKPHISL RAALRDNFRM KFFYLHLSYL KKAIDLEGAN
VKGYFAWSLT DNFEWNYGYT IRFGIIYVDH NNFARYKKLS AVWFKRFLK

Fig. S6 continued.

TRINITY_DN1383_c0_g1_i2 5 (6% relative abundance, 15 peptides, 15 unique peptides)
homolog to LRR receptor-like serine/threonine protein kinase GSO1 (C0LGQ5), identities: 105/324 (32%)

```
FFFPISKFTM TQPRPSFFFF FLFPFILHLI FLFFASSVQS LTLYSDIEAL KSFRASIKTT
SIPSYSCLGS WNFTAADPCA NPRVTYFTCG LMCNGNRVTH ITLDPAGYVG TLTPLISKLT
QLIALDLSTN QFRGPIPVIS SLTNLQTLVL RSNSFSGGIP PSLTALKSLE TLDFSHNYLS
GALPKSMNSL VSLIRLDLSF NRLAGPLPKL PPNIIELAIK ANSISGLLYK ASFDGLSRLE
VVELSENRLT GTIQPWFFLL PSLQQVDLAN NSFTGIEIWK SSGLNSDLVA VDLGFNKIEG
KLPVNFVSGG YPLLSSLSLR YNQFRGPIPV EYSKKETLKR LYLDGNYLNG WPPSGFFTGD
TSFSGSLGDN CLRNCPTSSQ LCLKSQKPAS ICQLAYGGKP RS
```

TRINITY_DN4175_c2_g1_i11 1 (6% relative abundance, 7 peptides, 7 unique peptides)
homolog to FLA1, fasciclin-like arabinogalactan protein 1 (Q9FM65), identities: 240/387 (62%)

```
RTFKSIYSLL LHSSLLILSF HSLPQISSSQ VTQPISKGKE SFPKNLCNHL LFHFHAFIMQ
LRTATVAAAV AFSFAFFLLP SATRAHNITH ILAGFPEFST FNHYLTTTHL AAEINSRETI
TVCAVDNAGM ADLLSKHLSI YALKNVLSLH VLLDYFGAKK LHQITNGTAL AATMYQATGS
APGSSGFVNI TDLKGGKVGF GAVDSGAIDA TFVKSIKEIP YNISIIQISK ILPSPDAEAP
TPGPSQMNLT SIMSAHGCKV FAETLLTSPA EKTYEDNVDG GLSVFCPGDD AMKNFLPKFK
NLTADGKQSL LEYHATPVYM SMPMLKSNNG PMNTLATDGP NKFAFVIQND GQEVTLKSKL
VTTKIISTLI DEQPLAIYSI NKVLLPKELF KGALAPTPAP APSPDAADAP SPKKSKKHKS
PPAPAAPADS PADGPAADSA DQTADGNFAV RFSGGRILAG ALSFWFAIFI L
```

Fig. S6 continued.

Table S1 The two gradients used for HPAEC-PAD analyses. Buffer A was 200 mM NaOH, buffer B was 15 mM NaOH and buffer C was 50 mM NaOH with 0.5 M NaAc. The time is given in minutes.

Gradient 1				Gradient 2				
Time	%A	%B	%C		Time	%A	%B	%C
-13	100	0	0		-13	100	0	0
-8	100	0	0		-8	100	0	0
-7	0	100	0		-7		100	0
12	0	100	0		9	0	100	0
12.1	0	60	40		9.1	5	85	10
22	0	60	40		23	0	60	40
22.1	100	0	0		25	0	60	40
24	100	0	0		26	0	100	0
25	0	100	0		28	0	100	0

Table S2 Binding of monoclonal antibodies to two samples of the *Ceropegia sandersonii* polysaccharide. The *C. sandersonii* polymer was spotted along with a few commercial polymers to estimate the amount and strength of binding to monoclonal antibodies. Out of 46 selected antibodies only two showed a sufficiently strong binding.

Legend: signal strength is reported; very strong (++); strong (+): weak (+/-) or no signal (-)

#TB Unknown polymer	#TC Unknown polymer	Polygal. Acid	Pectin esterified	RG I	Pectic galactan	Larch arabinogalactan	Linear arabinan	Probe name	Epitope
-	-	++	+	-	+/-	-	-	**2F4**	Ca2+ cross linked HG
-	-	++	+	-	-	-	-	**JIM5**	Homogalactauronan with a low DE
-	-	++	++	+/-	-	-	-	**JIM7**	Homogalactauronan with a high DE
-	-	++	+	-	++	-	-	**LM19**	Methylesterified homogalactauronan (low DE)
-	-	++	++	-	-	-	-	**LM20**	Methylesterified homogalactauronan (high DE)
+	+	+	+/-	++	+	-	-	**INRA-RU2**	Backbone of rhamnogalacturonan I (4 units)
-	-	+/-	+/-	++	+	-	-	**INRA-RU1**	Backbone of rhamnogalacturonan I (12 units)
-	-	+/-	+/-	+	+	-	-	**CCRC-M1**	Rhamnogalacturonan I/Me BSA complex
-	-	+/-	+/-	+	+	-	-	**CCRC-M13**	Rhamnogalacturonan I/Me BSA complex
-	-	-	-	-	++	++	-	**LM5**	(1→4)-β-D-galactan
-	-	-	-	-	-	+	++	**LM6**	(1→5)-α-L-arabinan
-	-	-	-	-	-	-	++	**LM13**	Linearised (1→5)-α-L-arabinan
-	-	-	-	+/-	-	-	+	**LM16**	(1→5)-α-L-arabinan, RG backbone
-	-	-	-	-	-	-	-	**LM8**	Xylogalacturonan
-	-	-	-	-	-	-	-	**LM15**	Xyloglucan (XXXG motif)
-	-	-	-	-	-	-	-	**LM24**	Xyloglucan (XLLG motiv, weak to XXLG)
-	-	-	-	-	-	-	-	**LM25**	Xyloglucan (XXXG, XXLG, XLLG motiv; also GGGGGG)
-	-	-	-	-	-	-	-	**LM10**	(1→4)-β-D-xylan (low-substituted)
-	-	-	-	-	-	-	-	**LM11**	(1→4)-β-D-xylan (low-substituted)/arabinoxylan)
-	-	-	-	-	-	-	-	**LM23**	(1→4)-β-D-xylan/xylogalacturonan
-	-	-	-	-	-	-	-	**BS-400-2**	(1→3)-β-D-glucan
-	-	-	-	-	-	-	-	**BS-400-3**	(1→3) (1→4)-β-D-glucan
-	-	-	-	-	-	-	-	**BS-400-4**	(1→4)-β-D-mannan
-	-	-	-	-	-	-	-	**LM21**	(1→4)-β-D-mannan/galactomannan/glucomannan
-	-	-	-	-	-	-	-	**LM22**	(1→4)-β-D-mannan/glucomannan
-	-	-	-	-	-	-	-	**JIM8**	AGP (Gal-rich)
+	+	-	-	+/-	+/-	+/-	-	**JIM13**	AGP; β-GlcA-(1→3)-α-GalA-(1→2)-α-Rha
-	-	-	-	-	-	-	-	**LM2**	AGP; (1→6)-β-Gal with terminal β-GlcA
-	-	-	-	-	-	-	-	**LM14**	AGP and/or pectic type II arabinogalactan
-	-	-	-	-	-	-	-	**MAC207**	AGP; β-GlcA-(1→3)-α-GalA-(1→2)-α-Rha
-	-	-	-	-	-	-	-	**LM1**	Extensin (hydroxyproline-rich motif THRGP)
-	-	-	-	-	-	-	-	**LM3**	Extensin
-	-	-	-	-	-	-	-	**JIM11**	Extensin
-	-	+/-	+/-	-	-	-	-	**JIM12**	Extensin
-	-	-	-	-	-	-	-	**JIM19**	Extensin
-	-	+/-	+/-	-	-	-	-	**JIM20**	Extensin
-	-	-	-	-	-	-	-	**B-1005**	Concanavalin A; α-linked Man
-	-	-	-	-	-	-	-	**B-1015**	Soybean agglutinin; α- or β-linked GalNAc
-	-	-	-	-	-	-	-	**B-1025**	Wheat germ agglutinin; dimers and trimers of GlcNAc
-	-	-	-	-	-	-	-	**B-1065**	Ulex Europaeus Agglutinin I; α-linked Fuc
-	-	-	-	-	-	-	-	**B-1085**	Ricinus communis agglutinin; Gal or GalNAc residues
-	-	-	-	-	-	-	-	**B-1285**	Bauhinia purpurea lectin; Gal-α-(1→3) GalNAc, terminal GalNAc
-	-	-	-	-	-	-	-	**B-1305**	Sambucus nigra lectin; sialic acid attached to term. Gal, GalNAc
+/-	+/-	-	-	-	-	-	-	**B-1335**	Euonymus europaeus lectin; Gal-α-(1→3)-Fuc-α-(1→2)-Gal
-	-	-	-	-	-	-	-	**B-1365**	Psophocarpus tetragonolobus lectin; GalNAc
-	-	-	-	-	-	-	-	**B-1405**	Psophocarpus tetragonolobus lectin II; Gal and GalNAc

Table S3 Observed chemical shifts of the disaccharide GlcAβ1,6Gal at 298 K, referenced to DSS in comparison with chemical shift predictions with the software CASPER (Furevi *et al.*, 2022) and the NMR values of Menestresina et al. 1998 (Menestrina *et al.*, 1998).

Moiety	H1	H2	H3	H4	H5	H6	H6'	C1	C2	C3	C4	C5	C6	
GlcAβ1,6-	*4.545 (α)*[a]<br>*4.552 (β)*[a]	*3.36*	*3.54*	*3.55*	*3.85*[b]			*105.2*	*75.6*	*78.1*	*74.3*	*78.1*[b]	*177.1*[b]	this work
	4.52 (α)[a]<br>*4.54 (β)*[a]	*3.36*	*3.53*	*3.52*	*3.73 (α)*[a]<br>*3.72 (β)*[a]	—	—	*105.2*	*75.6*	*78.2*	*74.4*	*78.7*	*178.2*	CASPER[c]
	4.48 (α)[a]<br>*4.49 (β)*[a]							*105.2*					177.8	Menestresina[d]
-β1,6Galα	*5.28*	*3.81*	*3.87*	*4.02*	*4.28*	*3.84*	*4.06*	*95.0*	*71.0*	*71.7*	*72.0*	*71.9*	*72.1*	this work
	5.22	*3.77*	*3.82*	*3.98*	*4.22*	*3.82*	*3.98*	*95.0*	*71.0*	*71.7*	*72.0*	*71.8*	*71.5*	CASPER[c]
	5.23							*95.0*						Menestresina[d]
-β1,6Galβ	*4.60*	*3.51*	*3.66*	*3.96*	*3.90*	*3.88*	*4.06*	*99.1*	*74.5*	*75.3*	*71.5*	*77.8*	*72.0*	this work
	4.55	*3.46*	*3.60*	*3.93*	*3.84*	*3.79*	*4.00*	*99.0*	*74.6*	*75.4*	*71.5*	*76.4*	*71.4*	CASPER[c]
	4.55							*99.1*						Menestresina[d]

[a] the chemical shifts depend on the anomeric form at the Gal at the reducing end. (α) stands for Galα at the reducing end and (β) stands for Galβ at the reducing end.

[b] the chemical shift values are pH dependent due to the equilibrium between COOH and COO^- at C6

[c] the calculated ^{13}C values showed an offset due to different referencing. For better comparison a correction of +1.7 ppm was added to the ^{13}C values

[d] the calculated ^{13}C values showed an offset due to different referencing. For better comparison a correction of +2.2 ppm was added to the ^{13}C values

Table S4 Observed chemical shifts of the intact polysaccharide of *Ceropegia sandersonii* referenced to DSS at 25°C in comparison with previously reported NMR data (Tan *et al.*, 2010; Cartmell *et al.*, 2018; Tan *et al.*, 2023). In addition very small signals of short Ara*f*α1,3Galβ1,6- and Galβ1,6-side chains are also visible in the spectra but omitted here. Their chemical shifts are reported in Table S5. The name of the species in the references is indicated in brackets.

Comment	Moiety	H1	H2	H3	H4	H5	H6/ H5'	H6'	C1	C2	C3	C4	C5	C6	
linear side chain<br><br>Rhaα1,4 GlcAβ1,6 Galβ1,6- bbGal	Rha (b)	*4.747*	*3.952*	*3.781*	*3.436*	*4.043*	*1.262*		*103.4*	*72.9*	*72.8*	*74.6*	*71.7*	*19.2*	this work
		4.742	*3.926*	*3.746*	*3.432*	*4.013*	*1.245*	—	*103.6*[a]	*72.9*[a]	*72.8*[a]	*74.6*[a]	*71.8*[a]	*19.1*[a]	Cartmell
		4.90	*3.98*	*3.75*	*3.47*	*3.96*	*1.26*		*103.6*[b]	*73.2*[b]	*73.1*[b]	*74.8*[b]	*71.9*[b]	*19.2*[b]	CASPER
	GlcA (d)	*4.525*	*3.384*	*3.584*	*3.59*	*3.763*	—	—	*105.3*	*76.0*	*76.9*	*81.8*	*78.8*	*177.9*	this work
		4.558	*3.369*	*3.605*	*3.639*	*3.955*	—	—	*105.4*[a]	*75.9*[a]	*76.8*[a]	*81.6*[a]	*77.1*[a]	*176.0*[a]	Cartmell
		4.55	*3.38*	*3.65*	*3.66*	*3.80*	—	—	*105.2*[b]	*76.0*[b]	*77.1*[b]	*82.4*[b]	*78.1*[b]	*178.0*[b]	CASPER
	Gal (f)	*4.459*	*3.548*	*3.669*	*3.965*	*3.93*	*4.076*	*3.917*	*106.3*	*73.4*	*75.3*	*71.3*	*76.2*	*72.3*	this work
		4.433	3.529	3.651	3.934	3.894	4.016	3.897	*105.8*[a]	*73.4*[a]	*75.2*[a]	*71.3*[a]	*76.5*[a]	*72.2*[a]	Cartmell
		4.44	*3.52*	*3.61*	*3.91*	*3.84*	*4.02*	*3.81*	*105.9*[b]	*73.7*[b]	*75.6*[b]	*71.7*[b]	*76.6*[b]	*71.7*[b]	CASPER
branched side chain<br><br>Rhaα1,4 GlcAβ1,6 [Ara*f*α1,3] Galβ1,6- bbGal [c]	Rha (b)	*4.747*	*3.952*	*3.781*	*3.436*	*4.043*	*1.262*	—	*103.4*	*72.9*	*72.8*	*74.6*	*71.7*	*19.2*	this work
		4.81	*3.95*	*3.78*	*3.42*	*4.02*	*1.25*	—	*103.3*[d]	*73.0*[d]	*73.0*[d]	*74.8*[d]	*71.7*[d]	*19.2*[d]	Tan 2010 (R)
	GlcA (d)	*4.525*	*3.384*	*3.584*	*3.59*	*3.763*	—	—	*105.3*	*76.0*	*76.9*	*81.8*	*78.8*	*177.9*	this work
		4.51	*3.45*	*3.56*[e]	*3.63*	*3.73*[e]	—	—	*105.3*[d]	*76.0*[d]	*77.1*[d,e]	*81.8*[d]	*79.0*[d,e]		Tan 2010 (UA)
	Araf (a)	*5.25*	*4.232*	*3.948*	*4.138*	*3.841*	*3.715*	—	*111.9*	*84.0*	*79.3*	*86.5*	*64.0*	—	this work
		5.25	*4.21*	*3.95*	*4.13*	*3.82*	*3.71*	—	*111.9*[d]	*83.9*[d]	*79.3*[d]	*86.7*[d]	*64.0*[d]	—	Tan 2010 (A)
	Gal (e)	*4.505*	*3.658*	*3.732*	*4.152*	*3.914*	*4.076*	*3.917*	*106.1*	*72.5*	*82.9*	*71.1*	*76.3*	*72.3*	this work
		4.47	*3.65*	*3.71*	*4.11*	*3.91*	*4.04*	*3.94*	*106.1*[d]	*73.3*[d]	*83.3*[d]	*71.5*[d]	*76.3*[d]	*72.1*[d]	Tan 2010 (Ga)
short linear side chain<br><br>GlcAβ1,6 Galβ1,6- bbGal	GlcA (d′)	*4.522*	*3.358*	*3.531*	*3.536*	*3.742*	—	—	*105.3*	*75.6*	*78.2*	*74.5*	*78.6*	*n.d.*	this work
		4.51	*3.34*	*3.52*[e]	*3.54*	*3.74*[e]	—	—	*105.8*[f]	*75.7*[f]	*78.0*[e,f]	*73.0*[f]	*78.2*[e,f]	*n.r.*	Tan 2023 (E)
		4.54	*3.36*	*3.54*	*3.52*	*3.72*	—	—	*105.4*[b]	*75.8*[b]	*78.5*[b]	*74.6*[b]	*78.9*[b]	*178.4*[b]	CASPER
	Gal (f)	*4.459*	*3.548*	*3.669*	*3.965*	*3.93*	*4.076*	*3.917*	*106.3*	*73.4*	*75.3*	*71.3*	*76.2*	*72.3*	this work

		4.46	*3.66*	*3.72*	*4.10*	*3.88*	*4.03*	*3.92*	*106.0*[f]	*72.2*[f]	*82.9*[f]	*71.1*[f]	*76.2*[f]	*72.0*[f]	Tan 2023 (F)
		4.44	*3.52*	*3.61*	*3.91*	*3.84*	*4.02*	*3.81*	*105.9*[b]	*73.7*[b]	*75.6*[b]	*71.7*[b]	*76.6*[b]	*71.7*[b]	CASPER
backbone Galβ1,3-	Gal (c)	*4.713*	*3.785*	*3.895*	*4.248*	*3.894*	*4.052*	*3.984*	*106.4*	*72.8*	*84.3*	*71.2*	*76.3*	*72.0*	this work
		4.64[g]	*3.65*[g]	*3.85*	*4.23*	*3.77*	*4.04*	*3.94*	*106.6*[d]	*73.2*[d]	*84.6*[d]	*71.3*[d]	*76.4*[d]	*72.1*[d]	Tan 2010 (G2)
		4.70	*3.79*	*3.88*	*4.21*	*3.79*	*4.03*	*3.92*	*106.3*[f]	*72.2*[f]	*84.8*[f]	*71.2*[f]	*75.9*[f]	*72.0*[f]	Tan 2023 (C)
		4.62	*3.73*	*3.78*	*4.19*	*3.86*	*3.99*	*3.85*	*106.8*[b]	*72.8*[b]	*84.8*[b]	*71.1*[b]	*76.2*[b]	*71.4*[b]	CASPER

[a] the reported ^{13}C values showed an offset due to different referencing. For better comparison a correction of +1.9 ppm was added to the ^{13}C values

[b] the calculated ^{13}C values showed an offset due to different referencing. For better comparison a correction of +1.9 ppm was added to the ^{13}C values

[c] CASPER does not include arabinofuranose and therefore the chemical shifts of oligosaccharides containing Ara*f* cannot be predicted

[d] the reported ^{13}C values showed an offset due to different referencing. For better comparison a correction of +1.6 ppm was added to the ^{13}C values

[e] the reported values seemed to have interchanged C3/H3 and C5/H5 assignments. Here we swapped the assignments

[f] the reported ^{13}C values showed an offset due to different referencing. For better comparison a correction of +2.0 ppm was added to the ^{13}C values

[g] values deviate, but values of Tan et al. 2023 fit

Table S5 Observed chemical shifts of the polysaccharide of *Ceropegia sandersonii* after enzymatic cleavage referenced to DSS at 25°C in comparison with previously reported NMR data (Delgobo *et al.*, 1999; Endo *et al.*, 2013) and predictions with the software CASPER (Furevi *et al.*, 2022). The compound number of the reference is indicated in brackets.

Comment	Moiety	H1	H2	H3	H4	H5	H6/ H5'	H6'	C1	C2	C3	C4	C5	C6	
linear side chain<br>Ara*f*α1,3 Galβ1,6-bbGal [a]	Araf (a)	*5.253*	*4.228*	*3.953*	*4.135*	*3.84*	*3.721*	—	*111.9*	*84.1*	*79.3*	*86.6*	*64.0*	—	this work
		5.26	*4.20*	*3.93*	*4.07*	*3.82*	*n.r.*	—	*111.7*[b]	*84.0*[b]	*79.3*[b]	*86.8*[b]	*63.9*[b]	—	Delgobo (4)
		5.25[c]	*4.22*[c]	*3.96*[c]	*n.r.*	*3.85*[c]	*3.76*[c]	—	*112.0*[d]	*84.0*[d]	*79.3*[d]	*86.6*[d]	*64.0*[d]	—	Endo
	Gal (e′)	*4.499*	*3.653*	*3.724*	*4.111*	*3.713*	*3.774*		*106.1*	*72.7*	*83.0*	*71.3*	*77.7*	*63.7*	this work
		4.48	*3.58*	*3.78*	*4.01*	*n.r.*	*n.r.*		*105.7*[b]	*73.7*[b]	*75.8*[b,e]	*77.7*[b,f]	*n.r.*	*64.0*[b]	Delgobo (4)
		4.48[c]	*n.r.*	*n.r.*	*n.r.*	*n.r.*	*3.80*[c]		*105.9*[d]	*n.r.*	*82.9*[d]	*70.6*[d]	*n.r.*	*n.r.*	Endo
single saccharide side chain Galβ1,6-bbGal	Gal (f′)	*4.449*	*3.548*	*3.653*	*3.928*	*3.706*	*3.776*		*106.3*	*73.5*	*75.5*	*71.4*	*77.8*	*63.7*	this work
		4.42	*3.51*	*3.60*	*3.87*	*3.65*	*3.74*	*3.66*	*106.0*[g]	*73.7*[g]	*75.7*[g]	*71.6*[g]	*77.9*[g]	*63.8*[g]	CASPER
backbone Galβ1,3-	Gal (c)	*4.713*	*3.786*	*3.882*	*4.241*	*3.931*	*4.057*	*3.92*	*106.5*	*73.0*	*84.5*	*71.3*	*76.2*	*72.0*	this work
		4.62	*3.73*	*3.78*	*4.19*	*3.86*	*3.99*	*3.85*	*106.8*[g]	*72.8*[g]	*84.8*[g]	*71.1*[g]	*76.2*[g]	*71.4*[g]	CASPER

[a] CASPER does not include arabinofuranose and therefore the chemical shifts of oligosaccharides containing Ara*f* cannot be predicted

[b] the reported ^{13}C values showed an offset due to different referencing. For better comparison a correction of +2.3 ppm was added to the ^{13}C values

[c] the reported ^{1}H values showed an offset due to different referencing. For better comparison a correction of +0.06 ppm was added to the ^{1}H values

[d] the reported ^{13}C values showed an offset due to different referencing. For better comparison a correction of +2.6 ppm was added to the ^{13}C values

[e] the reported value for C3 is likely incorrectly assigned

[f] the reported value for C4 is likely incorrectly assigned, it would fit to C5

[g] the calculated ^{13}C values showed an offset due to different referencing. For better comparison a correction of +1.9 ppm was added to the ^{13}C values

Table S6 Observed chemical shifts of the cleaved disaccharide Rhaα1,4GlcA at 298 K, referenced to DSS in comparison with chemical shift values of Fügedi 1987 (Fugedi, 1987) and predictions with the software CASPER (Furevi *et al.*, 2022).

Moiety	H1	H2	H3	H4	H5	H6	C1	C2	C3	C4	C5	C6	
Rhaα1,4 (b)	*4.727 (α)[a]<br>4.734 (β)[a]*	*3.953 (α)[a]<br>3.980 (β)[a]*	*3.784 (α)[a]<br>3.765 (β)[a]*	*3.441 (α)[a]<br>3.435 (β)[a]*	*4.052 (α)[a]<br>4.042 (β)[a]*	*1.260*	*103.5*	*73.0*	*72.8*	*74.7*	*71.6*	*19.20*	this work
	n.r.[b]	*n.r.[b]*	*n.r.[b]*	*n.r.[b]*	*n.r.[b]*	*n.r.[b]*	*103.6[c]*	*72.8[c]*	*72.8[c]*	*74.5[c]*	*71.8[c]*	*19.1[c]*	Fügedi
	4.90	*3.98*	*3.75*	*3.47*	*3.96*	*1.26*	*103.6[d]*	*73.2[d]*	*73.1[d]*	*74.8[d]*	*71.9[d]*	*19.2[d]*	CASPER
GlcAα (gα)	*5.239*	*3.611*	*3.743*	*3.548*	*4.137*	—	*94.7*	*74.4*	*74.2*	*82.4[e]*	*74.6[e]*	*n.d.*	this work
	n.r.[b]	*n.r.[b]*	*n.r.[b]*	*n.r.[b]*	*n.r.[b]*	—	*94.8[c]*	*73.9[c]*	*73.9[c]*	*81.4[c]*	*72.3[c]*	*n.r.[b]*	Fügedi
	5.25	*3.61*	*3.87*	*3.67*	*4.16*	—	*94.7[d]*	*74.5[d]*	*74.1[d]*	*82.6[d]*	*73.5[d]*	*178.9[d]*	CASPER
GlcAβ (gβ)	*4.633*	*3.311*	*3.559*	*3.576*	*3.759*	—	*98.6*	*77.1*	*77.1*	*82.0[e]*	*79.2[e]*	*n.d.*	this work
	n.r.[b]	*n.r.[b]*	*n.r.[b]*	*n.r.[b]*	*n.r.[b]*	—	*98.7[c]*	*76.7[c]*	*76.7[c]*	*81.7[c]*	*76.2[c]*	*n.r.[b]*	Fügedi
	4.66	*3.32*	*3.64*	*3.68*	*3.80*	—	*98.5[d]*	*77.2[d]*	*77.1[d]*	*82.4[d]*	*78.0[d]*	*177.9[d]*	CASPER

[a] the chemical shifts depend on the anomeric form at the Gal at the reducing end. (α) stands for GlcAα at the reducing end, (β) stands for GlcAβ at the reducing end

[b] n.r. stands for not reported, few ^{1}H data were reported for a different solvent

[c] the reported ^{13}C values showed an offset due to different referencing. For better comparison a correction of +1.9 ppm was added to the ^{13}C values

[d] the predicted ^{13}C values showed an offset due to different referencing. For better comparison a correction of +1.9 ppm was added to the ^{13}C values

[e] the atoms are close to the carboxyl group which is in equilibrium between the neutral and charged form depending on the pH. Chemical shifts in the environment can change depending on the pH value.

Methods S1

Efforts to identify the protein component of the Ceropegia polymer by LC-HRMS

One 110 µL aliquot of the gliding zone wash-off was diluted 1/2 (v/v) in 100 mmol/L triethylammonium bicarbonate buffer (TEAB, pH 8.50, Sigma-Aldrich), containing 10 % (w/w) sodium dodecyl sulfate (SDS, Sigma-Aldrich) and 1× protease inhibitor cocktail (Roche), and heated for 5 min at 95°C for denaturation. In order to reduce possible disulfides, the sample was treated with 5 mmol/L tris-(2-carboxyethyl)-phosphine-hydrochloride (TCEP, Sigma-Aldrich) at 55 °C for 15 min. This was followed by alkylation of the cysteine residues by addition of iodoacetamide (Sigma-Aldrich) to a final concentration 40 mmol/L and incubation at 22 °C in the dark for 10 min. After this, the sample was acidified to pH ≤1 with 12 % (v/v) ortho-phosphoric acid (Merck) followed by protein precipitation by adding 7:1 (v/v) of 100 mM TEAB (pH 7.55) in 90 % methanol (v/v). Next the proteins were purified by suspension trapping employing S-Trap mini columns (Protifi) according to the manufacturer's instructions,and digested to peptides by addition of 10 µg trypsin (sequencing grade modified, porcine, Promega, Madison, WI, USA) and incubation at 37 °C for 16 h. The purified peptides were dried at 50 °C in a vacuum centrifuge and resuspended in 10 µL 1.0% aqueous acetonitrile (ACN; VWR International) with 0.010% formic acid (FA; Sigma-Aldrich). Next, 0.330 µL of the sample were injected and chromatographically separated by reversed phase HPLC on an UltiMate™ 3000 RSLCnano System (Thermo Fisher Scientific), employing a DNV PepMap™ Neo column (150 x 0.075 mm i.d.) (Thermo Fisher Scientific). For the separation, 0.10 % aqueous FA (solvent A) and 0.10 % FA in ACN (solvent B) were pumped at a flow rate of 300.0 nL/min in the following order: 1.0 % B for 2.0 min, a linear gradient from 1.0-10.0 % B in 3.0 min, a second linear gradient from 10.0-35.0 % B in 45.0 min, and a third linear gradient from 35.0-45.0 % B in 10.0 min. This was followed by flushing with 99.0 % B for 5 min and column re-equilibration with 1.0 % B for 30 min. The column temperature was kept constant at 50 °C, the autosampler was kept at 5 °C. The nanoHPLC system was hyphenated to a Q Exactive™ Hybrid Quadrupole-Orbitrap™ mass spectrometer via a Nanospray Flex™ ion source (both from Thermo Fisher Scientific). The source was equipped with a SilicaTip emitter with 360 µm o.d., 20 µm i.d. and a tip i.d. of 10 µm (CoAnn Technologies Inc). The spray voltage was set to 1.5 kV, S-lens RF level

to 60.0 and capillary temperature to 250 °C. Each scan cycle consisted of a full scan at a scan range of m/z 350–2,000 and a resolution setting of 70,000 at m/z 200, followed by 15 data-dependent higher-energy collisional dissociation (HCD) scans in a 2.0 m/z isolation window at 28 % normalized collision energy at a resolution setting of 17,500 at m/z 200. For the full scan the automatic gain control (AGC) target was set to 3e6 charges with a maximum injection time of 100 ms, for the HCD scans the AGC target was 1e5 charges with a maximum injection time of 150 ms. Already fragmented precursor ions were excluded for 30 seconds. Data acquisition was conducted using Thermo Scientific™ Chromeleon™ 7.2 CDS (Thermo Fisher Scientific). For raw data evaluation, MaxQuant 2.0.1.0 (Cox & Mann, 2008) was used in the default settings. For protein identification a custom protein sequence database was created by translating all Ceropegia transcriptome sequences in all six reading frames using transeq (Madeira *et al.*, 2022). Subsequently, the longest continuous protein sequence fragment appearing within the respective six reading frames was extracted and added to the custom database. This custom-built database was used for protein identification, applying a 1 % false discovery rate. The mass spectrometry proteomics data have been deposited to the ProteomeXchange Consortium via the PRIDE (Perez-Riverol *et al.*, 2025) partner repository with the dataset identifier PXD062347.

References:

Cartmell A, Muñoz-Muñoz J, Briggs JA, Ndeh DA, Lowe EC, Baslé A, Terrapon N, Stott K, Heunis T, Gray J, et al. 2018. A surface endogalactanase in *Bacteroides thetaiotaomicron* confers keystone status for arabinogalactan degradation. *Nature Microbiology* **3**(11): 1314-1326.

Cox J, Mann M. 2008. MaxQuant enables high peptide identification rates, individualized p.p.b.-range mass accuracies and proteome-wide protein quantification. *Nature Biotechnology* **26**(12): 1367-1372.

Delgobo CL, Gorin PAJ, Tischer CA, Iacomini M. 1999. The free reducing oligosaccharides of angico branco (*Anadenanthera colubrina*) gum exudate: an aid for structural assignments in the heteropolysaccharide. *Carbohydrate Research* **320**(3-4): 167-175.

Endo M, Kotake T, Watanabe Y, Kimura K, Tsumuraya Y. 2013. Biosynthesis of the carbohydrate moieties of arabinogalactan proteins by membrane-bound β-glucuronosyltransferases from radish primary roots. *Planta* **238**(6): 1157-1169.

Fugedi P. 1987. Synthesis of 4-O-(Alpha-L-Rhamnopyranosyl)-D-Glucopyranuronic Acid. *Journal of Carbohydrate Chemistry* **6**(3): 377-398.

Furevi A, Ruda A, D'Ortoli TA, Mobarak H, Ståhle J, Hamark C, Fontana C, Engström O, Apostolica P, Widmalm G. 2022. Complete ^{1}H and ^{13}C NMR chemical shift assignments of mono-to tetrasaccharides as basis for NMR chemical shift predictions of oligo- and polysaccharides using the computer program CASPER. *Carbohydrate Research* **513**: 108528.

Madeira F, Pearce M, Tivey ARN, Basutkar P, Lee J, Edbali O, Madhusoodanan N, Kolesnikov A, Lopez R. 2022. Search and sequence analysis tools services from EMBL-EBI in 2022. *Nucleic Acids Research* **50**(W1): W276-W279.

Menestrina JM, Iacomini M, Jones C, Gorin PAJ. 1998. Similarity of monosaccharide, oligosaccharide and polysaccharide structures in gum exudate of *Anacardium occidentale*. *Phytochemistry* **47**(5): 715-721.

Perez-Riverol Y, Bandla C, Kundu DJ, Kamatchinathan S, Bai J, Hewapathirana S, John NS, Prakash A, Walzer M, Wang S, et al. 2025. The PRIDE database at 20 years: 2025 update. *Nucleic Acids Research* **53**: D543-D553.

Tan L, Varnai P, Lamport DTA, Yuan CH, Xu JF, Qiu F, Kieliszewski MJ. 2010. Plant O-Hydroxyproline Arabinogalactans Are Composed of Repeating Trigalactosyl Subunits with Short Bifurcated Side Chains. *Journal of Biological Chemistry* **285**(32): 24575-24583.

Tan L, Xu JF, Held M, Lamport DTA, Kieliszewski M. 2023. Arabinogalactan Structures of Repetitive Serine-Hydroxyproline Glycomodule Expressed by *Arabidopsis* Cell Suspension Cultures. *Plants-Basel* **12**(5): 1036.
